# Digital Image Analysis of Picrosirius Red Staining: A Robust Method for Multi-Organ Fibrosis Quantification and Characterization

**DOI:** 10.3390/biom10111585

**Published:** 2020-11-22

**Authors:** Guillaume E. Courtoy, Isabelle Leclercq, Antoine Froidure, Guglielmo Schiano, Johann Morelle, Olivier Devuyst, François Huaux, Caroline Bouzin

**Affiliations:** 1 IREC Imaging Platform (2IP), Institut de Recherche Expérimentale et Clinique (IREC), Université Catholique de Louvain, 1200 Brussels, Belgium; guillaume.courtoy@uclouvain.be; 2 Laboratory of Hepato-Gastroenterology, Institut de Recherche Expérimentale et Clinique (IREC), Université Catholique de Louvain, 1200 Brussels, Belgium; 3 Pole of Pneumology, Institut de Recherche Expérimentale et Clinique (IREC), Université Catholique de Louvain, 1200 Brussels, Belgium; antoine.froidure@uclouvain.be; 4 Mechanisms of Inherited Kidney Diseases Group, University of Zurich, 8057 Zurich, Switzerland; guglielmo.schiano@uzh.ch (G.S.); olivier.devuyst@uzh.ch (O.D.); 5 Pole of Nephrology, Institut de Recherche Expérimentale et Clinique (IREC), Université Catholique de Louvain, 1200 Brussels, Belgium; johann.morelle@uclouvain.be; 6 Louvain Centre for Toxicology and Applied Pharmacology, Institut de Recherche Expérimentale et Clinique (IREC), Université Catholique de Louvain, 1200 Brussels, Belgium; francois.huaux@uclouvain.be

**Keywords:** fibrosis, picrosirius red, digital analysis, collagen proportionate area, whole section, region-of-interest, fibrosis pattern

## Abstract

Current understanding of fibrosis remains incomplete despite the increasing burden of related diseases. Preclinical models are used to dissect the pathogenesis and dynamics of fibrosis, and to evaluate anti-fibrotic therapies. These studies require objective and accurate measurements of fibrosis. Existing histological quantification methods are operator-dependent, organ-specific, and/or need advanced equipment. Therefore, we developed a robust, minimally operator-dependent, and tissue-transposable digital method for fibrosis quantification. The proposed method involves a novel algorithm for more specific and more sensitive detection of collagen fibers stained by picrosirius red (PSR), a computer-assisted segmentation of histological structures, and a new automated morphological classification of fibers according to their compactness. The new algorithm proved more accurate than classical filtering using principal color component (red-green-blue; RGB) for PSR detection. We applied this new method on established mouse models of liver, lung, and kidney fibrosis and demonstrated its validity by evidencing topological collagen accumulation in relevant histological compartments. Our data also showed an overall accumulation of compact fibers concomitant with worsening fibrosis and evidenced topological changes in fiber compactness proper to each model. In conclusion, we describe here a robust digital method for fibrosis analysis allowing accurate quantification, pattern recognition, and multi-organ comparisons useful to understand fibrosis dynamics.

## 1. Introduction

Fibrosis is a pathological manifestation of excessive or inappropriate wound healing resulting in collagen deposition that may affect the function of diverse organs [1]. So far, no effective anti-fibrotic therapy is available while the burden of fibrotic diseases is increasing and intense research on preclinical models is underway. Therefore, accurate and reliable quantification of collagen deposits and their characterization are necessary to stage and map the natural history of diseases, to study the pathogenesis of fibrosis, to identify therapeutic targets, to evaluate anti-fibrotic strategies or to validate non-invasive diagnostic tools.

Collagen biochemical assays (e.g., hydroxyproline measurements) on total tissue homogenate provide a global estimation of collagen levels within a tissue specimen. However, this approach faces multiples limitations: (1) techniques are somehow painstaking (hydroxyproline content or sodium dodecyl sulfate-polyacrylamide gel electrophoresis followed by densitometry analysis) or require purification steps to limit overestimation (Sircol) [2], carrying the risk of missing a significant component in discarded fractions; (2) they include both induced and structural collagen; (3) histological compartments cannot be distinguished, complicating data interpretation and hiding subtle changes [3].

Alternatively, histopathological parameters based on collagen staining on tissue sections have been validated in many organs and disease conditions for the grading and the staging of fibrosis or for follow-up purposes. In this approach, the first critical step is to provide a specific signal. The most common techniques to visualize collagen deposits are the Masson’s trichrome and picrosirius red (PSR) (Sirius red F3B, Direct red 80). Both dyes possess good affinity for collagen fibers owe to multiple base-acid interactions, with PSR having a higher binding affinity for fibrillary collagen because of its elongated chemical structure, as confirmed by immunodetection [4,5,6]. PSR staining offers numerous advantages: besides being inexpensive, PSR solution is stable for years, the staining protocol is fast and simple, consistent in terms of sensitivity and hue [5], and provides a contrasted staining that enables a more reliable evaluation of fibrosis than with the Masson’s trichrome [7,8,9]. Several protocols have been described for PSR staining, the most common being with yellow counterstaining by picric acid only which provides a good signal-to-noise ratio [4,10], with the use of additional dyes for specific applications [11,12] or combined with immunohistochemistry but with some limitations [13].

The staining interpretation represents the second critical step. It requires (1) the accurate and reliable detection of the signal over noise, (2) the identification of the relevant histological structures, and (3) a meaningful quantification. This can be achieved by skilled pathologists or using digital image analysis (DIA), both methods having intrinsic strengths and limitations. On the one hand, trained human brains can perform incredibly fast for completing scoring systems (i.e., Ishak, Metavir, or Ashcroft systems), but these human resources are usually limited and inter- and even intra-individual variations are a well-known source of error [14,15]. Furthermore, these scoring systems are by essence descriptive or only semi-quantitative, and comprehensive information from whole tissue PSR-stained sections remains under-exploited. On the other hand, modern DIA has benefited of the onset of artificial intelligence. Advanced algorithms have been developed to recognize some specific fibrosis patterns [16,17,18], but these methods require software that can be expensive and proper training. Furthermore, they are not intended for multi-organ comparison since their transposition would require specific adaptation and optimization. This can be solved by computer-assisted recognition of tissue architecture followed by automated quantification of fibrosis on large tissue samples [19,20,21,22], thereby outclassing visual scoring on selected fields of view. 

Outcomes generated by DIA can then be expressed as the area occupied by collagen out of the entire examined tissue section (referred to as collagen proportionate area, CPA). This accessible method is in use for fibrosis quantification in the liver [8,23,24,25,26,27], kidney [9,28,29], or lung [26,30]. Additionally, DIA combined with advanced microscopy (second harmonic generation) allows the morphometric segmentation of collagen fibers and hence discriminating aggregated and distributed fibers [31,32]. Nevertheless, to the best of our knowledge, no transposable method to quantify fibrosis in multiple organs and to characterize fibrosis dynamics on PSR-stained sections imaged in brightfield has been reported.

The present DIA method was developed to offer (1) a robust and versatile quantification that enables the regionalized analysis of fibrosis in diverse tissues for multi-organ or inter-study comparison and (2) objective parameters to characterize the pattern of collagen deposits by the comprehensive analysis on large tissue specimens. 

## 2. Materials and Methods

### 2.1. Murine Models of Fibrosis

We used in the present study recognized models of liver, kidney or lung fibrosis. All male mice were kept under standard laboratory conditions in 12:12 h light-dark cycles, and food and water were supplied ad libitum. Animal procedures were in accordance with the National Research Council Guide for the Care and Use of Laboratory Animals, in agreement with ARRIVE guidelines 2.0, and were approved by the Institutional Review Board of UCLouvain (liver model: 2012/UCL/MD/026; lung model: 2018/UCL/MD/012; kidney model: 2018/UCL/MD/015). In every model, control animals were compared with animals exposed to the fibrosis agent and sacrificed at two time points corresponding to intermediate and severe fibrosis. We analyzed n = 5 animals for each group and time point.

#### 2.1.1. Liver Fibrosis

Carbon tetrachloride (CCl_4_)-induced liver fibrosis is a classical and robust model of bridging fibrosis in rodent. CCl_4_ is specifically metabolized in ~50% of hepatocytes in the center of the liver lobule to induce an acute CYP2E1-dependent production of highly reactive tricholoromethyl free radicals, causing centro-lobular necrosis that triggers a wound healing response. When the insult is repeated, successive rounds of wound healing occur, causing peri-central fibrosis and progressively forming fibrotic bridges between vascular structures dissecting the parenchyma [33,34]. Fifteen 7-week-old C57BL/6J male mice were purchased from Janvier (Le-Genest-St-Ile, France). Ten mice received an intraperitoneal injection of CCl_4_ (800 μL/kg body weight in corn oil) three times a week to induce fibrosis. Among them, five mice were treated for 2 weeks to induce incomplete central bridges (named hereafter *2w CCl_4_* group) and five mice were injected for 7 weeks to yield severe bridging fibrosis (*7w CCl_4_* group) [33,34]. The remaining five mice received the vehicle only (*controls*). At the time of sacrifice, 48 h after the last injection, mice where anesthetized. The caudate and the left lateral liver lobes were dissected out, fixed with 4% formaldehyde for 24 h and embedded in paraffin.

#### 2.1.2. Lung Fibrosis

Pulmonary fibrosis is a well-known side effect of bleomycin treatment because the lung expresses extremely low levels of bleomycin hydrolase. In the presence of iron and oxygen, non-hydrolyzed bleomycin generates reactive oxygen species. Subsequent cytotoxic lipid peroxides and oxidized proteins cause tissue injury and parenchymal fibrosis [35]. Fifteen 8-week-old C57BL/6J female mice were purchased from Janvier (Le-Genest-St-Ile, France) and divided in three groups of five animals. In the first group, the mice received a single endotracheal instillation with 0.02 IU bleomycin in 50 µL saline and were sacrificed 28 days after instillation. The unique instillation of bleomycin (named after *BLM-acute*) is a model known to induce a rapid but transient fibrosis. In the second group, the mice underwent repeated endotracheal instillation with 0.02 IU once-a-week for 8 weeks to promote severe and persistent fibrosis [35,36] (*BLM-chronic*). The control group was instilled with saline solution only. At the time of sacrifice, mice were anesthetized by an intraperitoneal injection of 20 mg sodium pentobarbital (Certa, Braine-l’Alleud, Belgium). One lung was dissected out, fixed with 4% formaldehyde for 24 h and embedded in paraffin.

#### 2.1.3. Kidney Fibrosis

Kidney fibrosis is a primary dose-limiting side effect of antineoplastic treatment with cisplatin. Cisplatin is predominantly eliminated by the kidney, accumulating in the cells lining the proximal and more distal tubules. Intracellular accumulation of cisplatin generates reactive oxygen species, cytotoxicity, and DNA damage, causing a cortical fibrosis sparing the glomeruli [37]. Kidney fibrosis was generated using a well-established model of repeated low-dose cisplatin, as previously described [38]. Fifteen male FVB/n mice aged 8–10 weeks (Charles River, Brussels, Belgium) were administered a weekly intraperitoneal injection of low-dose cisplatin (7 mg/kg, Sigma-Aldrich, Overijse, Belgium) for 3 or 4 weeks (named after *3w CIS* and *4w CIS* groups) vs. normal saline only (*controls*). At the time of sacrifice, mice where anesthetized, one kidney was dissected out, fixed with 4% formaldehyde for 24 h and embedded in paraffin.

### 2.2. Tissue Preparation and Standardized Protocol for Picrosirius Red Staining

Protocol for PSR staining was performed as described [4,5] with recent optimization [10]. Briefly, 5 µm paraffin sections [20] were first deparaffinized and rehydrated. Slides were immersed in 1.0% phosphomolybdic acid solution for 2 min for pH acclimatization and yellow counterstaining [10], rinsed in water and then incubated in a saturated aqueous picric acid solution containing 0.1% Direct red 80 (Sigma #365548) for 2 h at room temperature. After a brief 2 min wash in 0.01 N HCL and an additional wash in water, slides were dehydrated and mounted with a Dako automated coverslipper. Tissue sections were digitalized using a SCN400 slide scanner (Leica Biosystems, Wetzlar, Germany) at 20× magnification.

### 2.3. Analysis Workflow

Whole tissue sections were analyzed using the image analysis tool Author™ version 2017.2 (Visiopharm, Hørsholm, Denmark) in order to (1) detect the tissue and assist the definition of histological compartments, (2) detect PSR staining, and (3) quantify this staining. These three steps are described hereafter.

#### 2.3.1. Tissue Recognition and Computer-Assisted Segmentation of Histological Compartments

In all models, the tissue section was automatically defined. Artifacts and regions-of-exclusion (ROEs, listed in Table 1) were then automatically discarded and manual correction applied when required (i.e., incomplete or incorrect automatic detection). Total tissue area was quantified. Then, computer-assisted delineation of regions-of-interest (ROIs) was performed for each tissue model. Details and illustrations are provided in Supplementary Material 1. Briefly,

1. For liver sections, connective tissue was classified as perivascular ROI when it directly surrounded a lumen (with or without erythrocytes) or as bridges when located in the parenchyma. The remaining tissue was considered as parenchyma.
2. For the lung, large connective tissue surrounding air ducts was discarded since its presence was highly variable between sections, introducing a bias. PSR staining was referred as peri-air ducts or perivascular ROIs when found at a maximum distance of 25 µm from air ducts (bronchi and bronchioles) or from blood vessels. The remaining tissue was considered as parenchyma.
3. For the kidney, the inner medulla, the outer medulla, and the cortex were manually defined based on morphological criteria, as described [39]. The cortex was further separated divided into large cortical vessels and vessel-free cortex.

**Figure 1:**
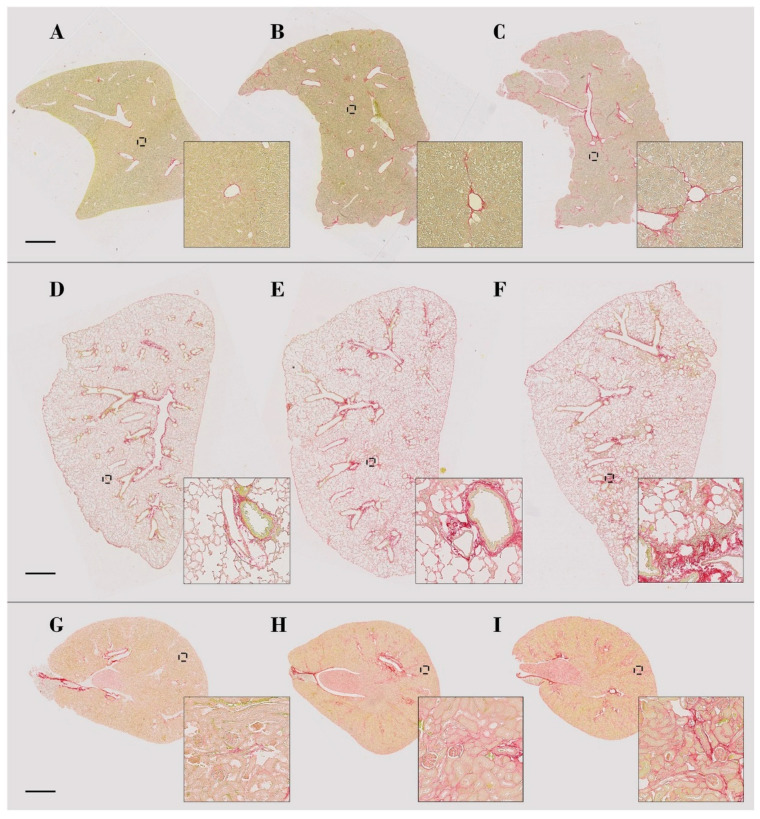
Illustration of fibrosis development in the murine models of liver (**A**–**C**), lung (**D**–**F**) and kidney (**G**–**I**) fibrosis. Representative samples of control (**A**), 2w CCl_4_ (**B**), and 7w CCl_4_ (**C**) livers; control (**D**), BLM-acute (**E**), and BLM-chronic (**F**) lungs; control (**G**), 3w CIS (**H**), and 4w CIS (**I**) kidneys. Global view of entire histological sections (scale bar = 1 mm) and higher magnification of specific regions (inserts).

ROIs and ROEs are illustrated in Figure 1. The respective proportion of the area of each ROI vs. total tissue area was calculated as

(1) $$\mathrm{Relative}\;\mathrm{ROI}\;\mathrm{area}\;=\;\frac{\Sigma \;Area_{\;selected\;ROI}}{Area_{\;total\;tissue}\;}\;\times \;100\;\left(in\;\%\right)$$

#### 2.3.2. Optimized Algorithm to Detect PSR-Stained Collagen Deposits and Fiber Segmentation 

Two detection methods for PSR staining were designed and illustrated in the Supplement Material 2. The first method (**PSR_RGB_**) extracted the red pixels of red-green-blue (RGB) filtering (RGB-G filter). For the second method optimized for increased selectivity and specificity (**PSR_OPT_**), images were preprocessed using a combination of RGB-G and H-AEC filters to generate an image of the noise filter. This noise was subtracted to red–green contrast filter providing a robust detection of the true signal. Chromaticity red filter completed this detection thanks to its high resolution. Based on this optimized detection, PSR-stained collagen fragments were subsequently segmented (**PSR_MORF_**) by watershed and object separation and then classified according to their intensity values and density into strongly stained and plain segments (compact fibers) or weakly stained and loose segments (scattered fibers), in a close manner as defined elsewhere [31]. For each detection method, one threshold was arbitrarily chosen on stained vs. non-stained regions of the three organs to allow multi-organ comparison, as explained in Supplementary Material 3.

#### 2.3.3. PSR-Stained Collagen Deposits Quantification Parameters

Collagen proportionate area (CPA) value is a morphometric measurement of signal density. Values were calculated as conventionally defined [9,23,24,25,26,28,29,30]. When performed on the entire tissue section, the measure was here referred to as *in toto* CPA [Equation (2)]. When tissue is segmented in multiple ROIs, additional information becomes available such as the distribution of all collagen among the different ROIs, defined here as collagen proportion [CP, Equation (3)], and the proportion of collagen detected in a specific ROI within total tissue, defined here as ROI CPA [Equation (4)]. These measurements were calculated as follows:

(2) $$In\;toto\;\mathrm{CPA}\;=\;\frac{stained\;area_{\;total\;tissue}}{area_{\;total\;tissue\;}\;}\;\times \;100\;{\left(in\;\%\right)}_{\;}$$

(3) $$\mathrm{Collagen}\;\mathrm{proportion}\;=\;\frac{\Sigma \;stained\;area_{\;selected\;ROI}}{stained\;area_{\;total\;tissue\;}\;}\;\times \;100\;\left(in\;\%\right)$$

(4) $$\mathrm{ROI}\;\mathrm{CPA}\;=\;\frac{\Sigma \;stained\;area_{\;selected\;ROI}}{area_{\;total\;tissue\;}\;}\;\times \;100\;\left(in\;\%\right)\;$$

The same parameters were applied for the quantification of compact and scattered fibers detected with the PSR_MORF_ method. Quantifications were performed at maximal resolution and raw values were expressed as µm^2^. 

**Figure 2:**
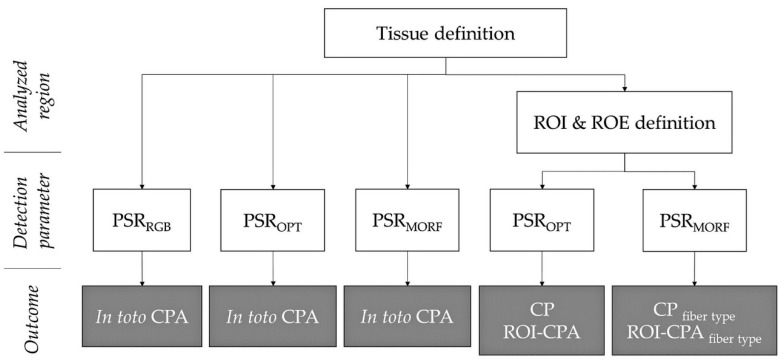
Analysis workflow for *in toto* vs. regionalized quantification using the three developed detection algorithms: RGB method (PSR_RGB_), optimized method (PSR_OPT_) or morphological fiber classification (PSR_MORF_). ROI, region-of-interest; ROE, region-of-exclusion; CPA, collagen proportionate area; CP, collagen proportion.

The analysis workflow is illustrated in Figure 2.

### 2.4. Statistics

Statistical analyses were performed using GraphPad Prism 8 on Windows10. One-way ANOVA were performed with Fisher’s post-hoc test. For correlation analysis, coefficient of determination (goodness-of-fit, R^2^) was computed with a confidence interval of 95%. For the fiber classification, inter-group comparisons were achieved by one-way ANOVA, while intra-group comparisons were computed by paired (CP) or unpaired (ROI CPA) *T*-tests. The respective control group for each model served for the multiple comparisons. *p*-value <0.05 was considered significant. Values are reported as the mean ± standard deviation (SD).

## 3. Results

### 3.1. Visual Examination of the Histological Sections

**Figure 3:**
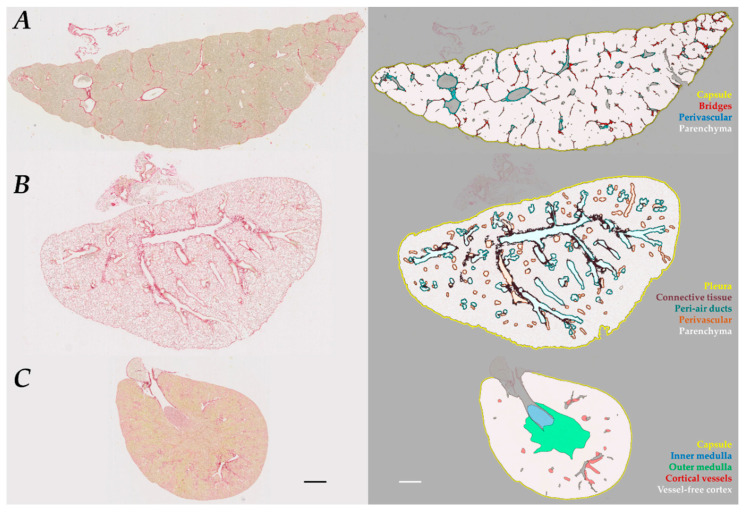
Computer-assisted segmentation of histological structures in the three studied organs. An illustration is given before (**left**) and after (**right**) semi-automated segmentation in (**A**) a 7w CCl_4_ liver, (**B**) a BLM-chronic lung, and (**C**) a 4w CIS kidney. Details in Supplementary Material 1. Scale bar = 1 mm.

As expected, specimen examination revealed that CCl_4_ exposure causes a peri-centrilobular liver fibrosis characterized by thickening of the vascular wall and progressive development of centro-central bridges in a time-dependent manner (Figure 3A). In the lungs, instillation of bleomycin induced alveolar hyperplasia (more extensive in the chronic model), patchy alveolar fibrosis, and inflammatory and/or fibroblastic foci often localized close to blood vessels (Figure 3B). Cisplatin injections led to the development of a time-dependent fibrosis around proximal and distal tubules in the inner layer of the renal cortex, as well as in the inner and outer medulla (Figure 3C).

### 3.2. A Robust and Tissue-Transposable Algorithm to Detect PSR Staining in Diverse Fibrotic Organs

**Figure 4:**
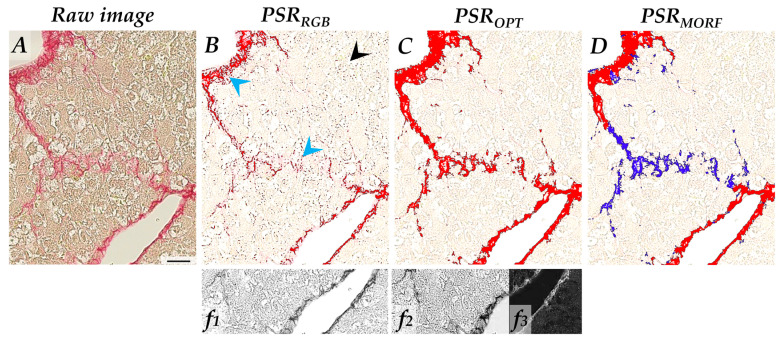
Detection methods of discrete liver fibrosis stained in PSR. (**A**) Native image. (**B**) PSR_RGB_ detection method, using RGB-G filter (insert ***f*1**). Staining detection is highlighted in red. Blue arrows: undetected pixels (false negative); black arrow: unsaturated pixels (false positive). (**C**) PSR_OPT_ detection method using optimized filters (inserts ***f*2** and ***f*3**). Staining detection is highlighted in red. (**D**) Optimized detection is used for morphological segmentation (based on fiber density and staining intensity) with algorithm PSR_MORF_. Compact fibers are labeled in red and scattered fibers are labeled in blue. Refer to Supplementary Materials 2 and 3A for the filters, the detailed procedure, and the dynamic ranges of sensitivity. Scale bar = 50 µm.

The first objective of the present study was to robustly detect PSR staining with an algorithm applicable to diverse organs. In first intention, we applied an RGB-based filtering to uncover PSR-stained pixels. As shown in Figure 4B, there is an apparent lack of sensitivity as fibers were incompletely detected (blue arrows). Moreover, despite a low detection threshold, non-PSR stained pixels were recognized as staining (Figure 4B, black arrow). These errors were more frequent in the livers than in lungs or kidneys.

To improve the specificity and sensitivity of the detection, we developed an optimized algorithm based on a more robust filtering of stained pixels (PSR_OPT_ method). This preprocessing increased the specificity for PSR staining vs. background, allowing lower detection threshold and more dynamic range, thereby also improving sensitivity of the detection (Figure 4C and Supplementary Material 2D).

**Figure 5:**
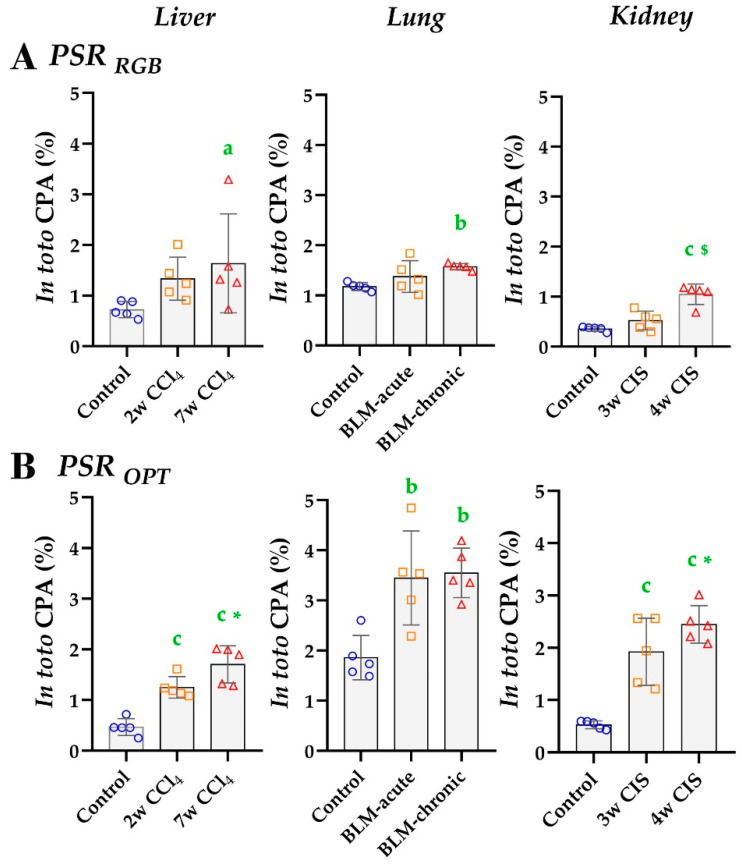
*In toto* CPA analyses using PSR_RGB_ (**A**) vs. PSR_OPT_ (**B**) filtering method for signal detection. Quantification performed on entire tissue sections (ROEs excluded). One-way ANOVA with Fisher’s post-hoc test. a: *p* < 0.050, b: *p* < 0.010, c: *p* < 0.001 vs. controls; *: *p* < 0.050, $: *p* < 0.001 last time point vs. intermediate time point.

The specificity and sensitivity of PSR_RGB_ and PSR_OPT_ detection methods were assessed by comparing *in toto* CPA values between organs at baseline and between the different time points within the same model. In accordance with the visual appraisal of staining at baseline (see Figure 3), *in toto* PSR_OPT_ CPA were ~4× higher in the control lungs than in the livers and the kidneys (Figure 5B and Supplementary Material 3B), and this difference was lower with the PSR_RGB_ method (Figure 5A). Comparing *in toto* CPA in the treated groups vs. their respective controls, PSR_RGB_ method evidenced fibrosis only at the latest time point of treatment in livers and kidneys and with chronic bleomycin treatment in the lungs (Figure 5A). By contrast, fibrosis at the early time point and progressive fibrosis were evidenced in the three organs using PSR_OPT_ (Figure 5B).

Correlation between the two methods was modest with a correlation coefficient weaker for the liver sections (0.499) as compared with the lungs (0.647) and the kidneys (0.693) (Supplementary Material 3C), tentatively explained by a higher noise in the liver fibrosis model.

These results suggest that the PSR_RGB_ detection method is not sensitive enough to highlight collagen deposits accumulation during fibrosis onset in these models, and this limitation could be circumvented, at least partially, by the PSR_OPT_ method.

### 3.3. Regionalized Analysis Revealed Topical Fibrosis Changes

The second aim was to evaluate whether regionalized analysis would allow a better characterization of fibrosis. To meet this goal, histologically relevant ROIs were defined for each organ (Figure 1 and Supplementary Material 1). The list of ROIs and ROEs and the relative area of ROIs are given in Table 1. PSR staining was then quantified in each ROIs using the PSR_OPT_ detection method. Results were expressed as CP (distribution of collagen among different ROIs) and ROI CPA (percentage of collagen detected in a specific ROI within total tissue).

#### 3.3.1. Liver Fibrosis

**Figure 6:**
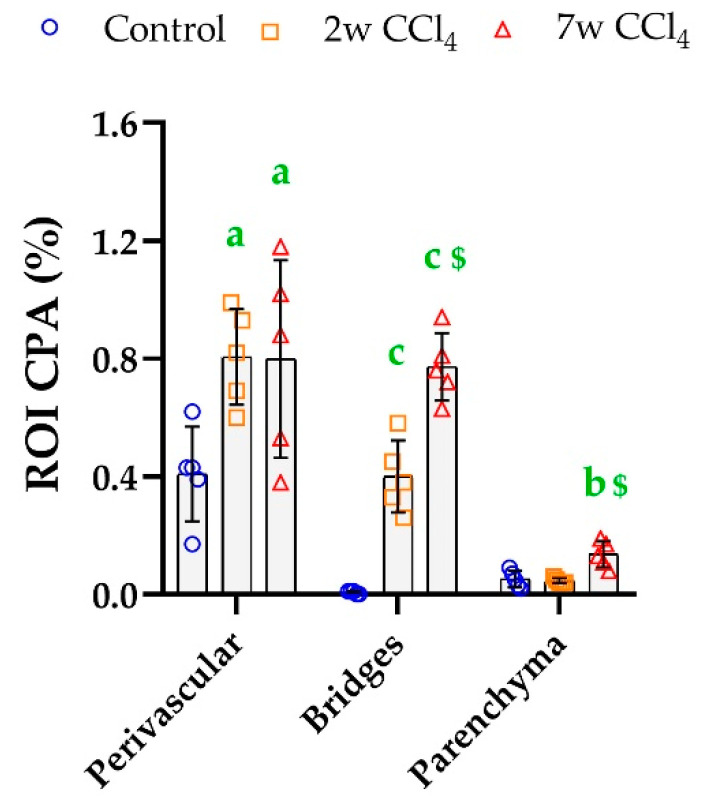
Liver ROI CPA calculated for each ROI using the PSR_OPT_ detection method. Mean (±SD) and one-way ANOVA with Fisher’s post-hoc test. a: *p* < 0.050, b: *p* < 0.010, c: *p* < 0.001 vs. controls; $: *p* < 0.001 vs. middle group.

While in normal livers, parenchyma represented more than 95% of the tissue sections (Table 1), PSR staining in this region only corresponded to ~10% of the total collagen amount (Table 2). The greatest CP was detected in the perivascular areas. This proportion decreased proportionally as the bridges formed (Table 2). Although the ROI CPA moderately increased around vascular structures in fibrotic livers (~2 times), this increase was much larger and time-dependent in bridges (~70 times at 2 weeks and ~130 times at 7 weeks). Collagen accumulation in the parenchyma and outside the fibrous septa was only detectable in the severe fibrotic condition (Figure 6).

#### 3.3.2. Lung Fibrosis

**Figure 7:**
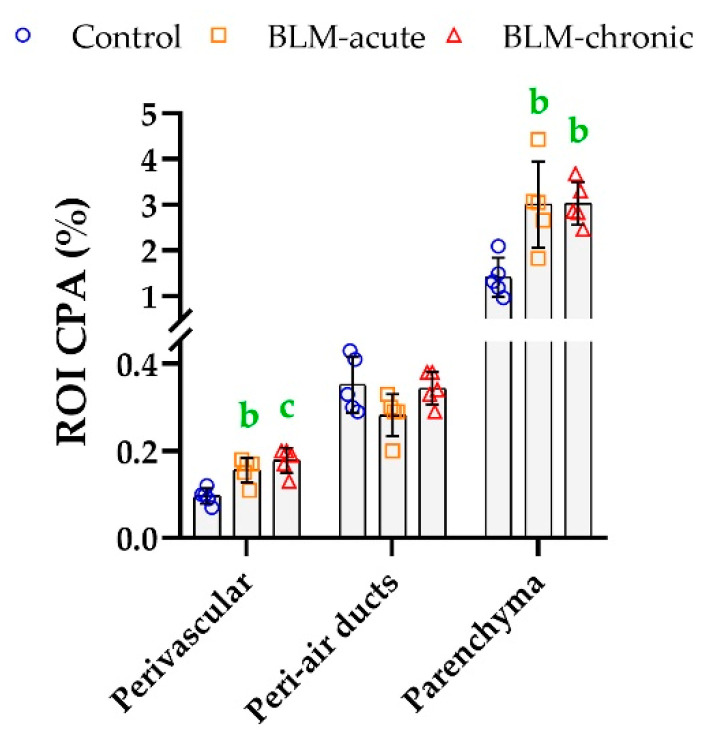
Lung ROI CPA calculated for each ROI using the PSR_OPT_ detection method. Mean (±SD) and one-way ANOVA with Fisher’s post-hoc test. b: *p* < 0.010, c: *p* < 0.001 vs. controls.

As shown in Table 1, the distribution of the ROIs (peri-air ducts, perivascular tissue, and parenchyma) was similar in the normal and bleomycin-treated tissues. In the healthy lung, the greatest CP was detected in the parenchyma (~75%), where it further increased after single or repeated bleomycin instillation (Table 3). The ROI CPA around air ducts was unchanged after fibrosis induction, but bleomycin caused a significant enrichment of PSR staining in the perivascular region (Figure 7).

#### 3.3.3. Kidney Fibrosis

**Figure 8:**
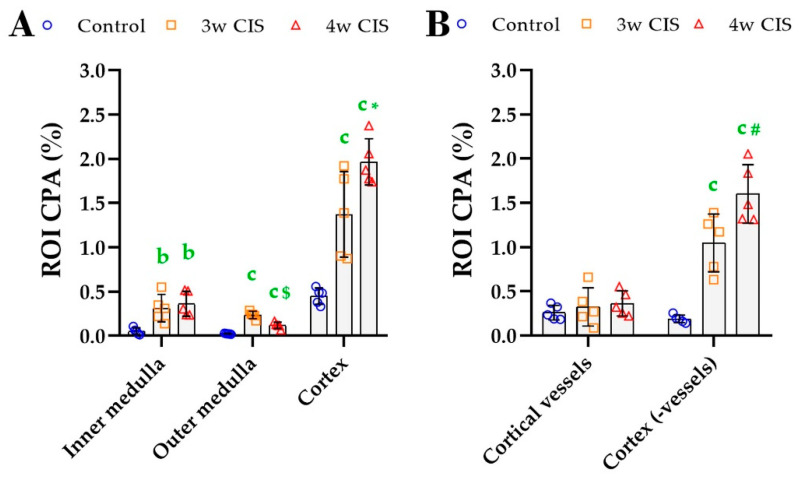
Kidney ROI CPA calculated using the PSR_OPT_ detection method for each ROI (**A**) and in sub-analyzing the cortex (**B**). Mean (±SD) and one-way ANOVA with Fisher’s post-hoc test. b: *p* < 0.010, c: *p* < 0.001 vs. controls; *: *p* < 0.050, ***#***: *p* < 0.010, $: *p* < 0.001 last time point vs. intermediate time point.

Under normal condition, the cortex was the dominant ROI (85% of the tissue) (Table 1) and the most important source of PSR signal (~85%); a large part of it was attributed to vessels (Table 4). Cisplatin treatment induced a reduction of the relative cortical area (Table 1), a time-dependent increase in the CP in the cortex (not attributed to vessels) and an elevation of the CP within the outer medulla (Table 4). The ROI CPA was increased in the inner medulla, outer medulla and cortex upon cisplatin administration (Figure 8A), with the largest accumulation in the (vessel-free) cortex (Figure 8B). The results of *in toto* CPA were validated independently, by using a previously described DIA method for PSR analysis [40] (Supplementary Material 4).

### 3.4. Morphological Parameters for Fibrosis Classification

Our third aim was to provide a tool for the automated categorization of collagen deposits based on the morphological organization of the fibers, as proposed. Using the same detection filter than PSR_OPT_, a new algorithm (PSR_MORF_) was developed to automatically segment the PSR fibers by watershed and separate objects, and quantify collagen fibers defined as compact or scattered based on the mean signal intensity of each segment (Figure 4D) (details given in Supplement Material 2B,D).

#### 3.4.1. *In Toto* Analysis of Fiber Morphology

**Figure 9:**
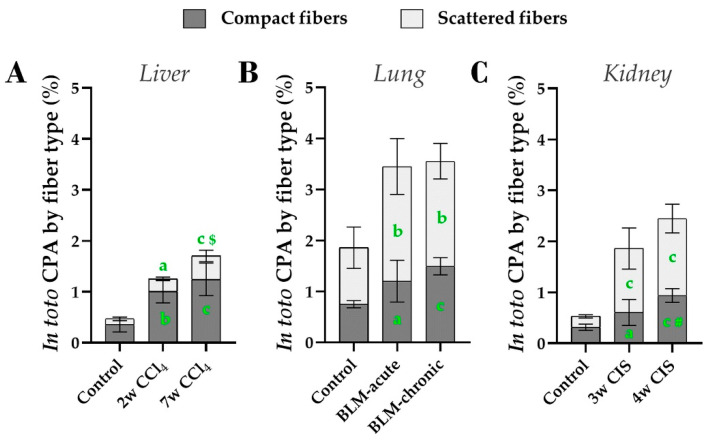
*In toto* analysis of compact and scattered fibers using PSR_MORF_ detection method, performed in total tissue for (**A**) the livers, (**B**) the lungs and (**C**) the kidneys. Mean (±SD) and one-way ANOVA with Fisher’s post-hoc test. a: *p* < 0.050, b: *p* < 0.010, c: *p* < 0.001 vs. controls; ***#***: *p* < 0.010, $: *p* < 0.001 last time point vs. intermediate time point.

Because the fiber classification by PSR_MORF_ was obtained from PSR_OPT_ algorithm, the sum of the two types of fibers calculated *in toto* is equal to the values described above (Figure 5 bottom panels). Compact fibers were dominant in control livers and kidneys, representing respectively ~75% and 60% of total collagen (Figure 9A,C), while the normal lung was proportionally richer in scattered fibers, (Figure 9B), at least after exclusion of the dense connective tissue surrounding air ducts as detailed above. Fibrosis in the liver was characterized by a proportional increase in both compact and scattered fibers. In contrast, bleomycin and cisplatin caused a preferential accumulation in scattered fibers in lungs and kidneys.

#### 3.4.2. Regionalized Analysis of Fiber Morphology

**Figure 10:**
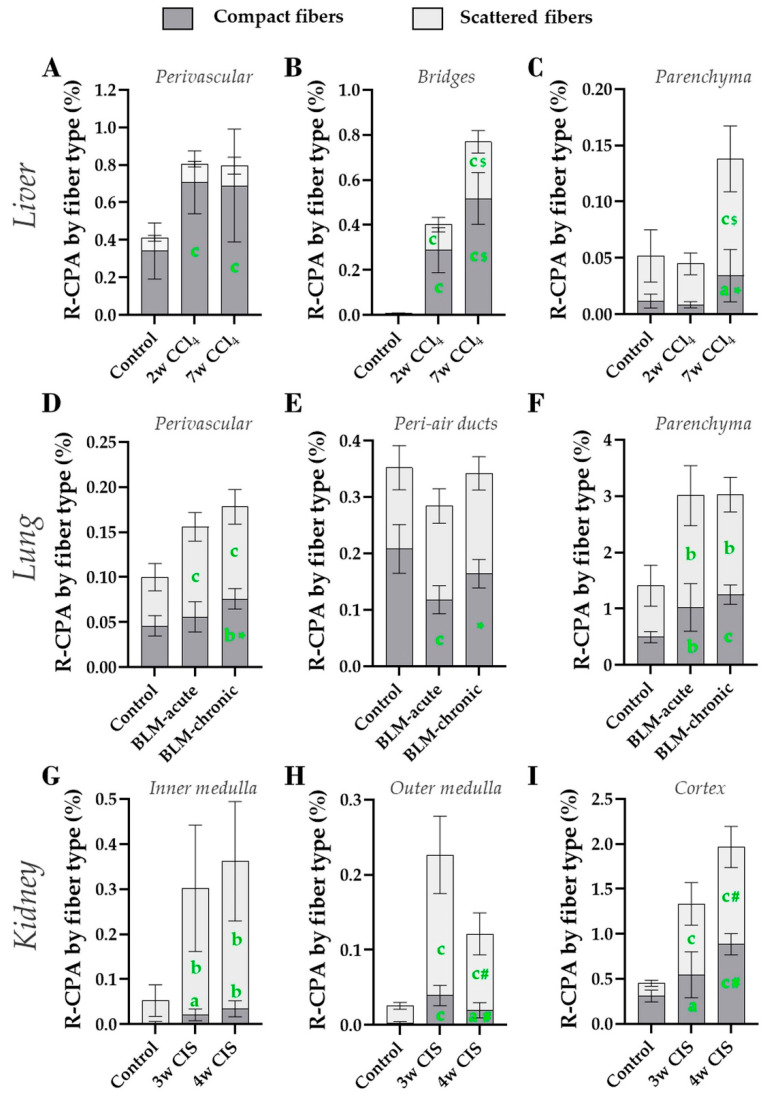
Regionalized analysis of compact and scattered fibers with PSR_MORF_ detection method. Results are shown in the liver for the perivascular region (**A**), bridges (**B**), and parenchyma (**C**), in the lung for the perivascular region (**D**), the peri-air duct region (**E**), and the parenchyma (**F**), and in the kidney for the inner medulla (**G**), the outer medulla (**H**) and the cortex (vessels included) (**I**). Mean (±SD). One-way ANOVA with Fisher’s post-hoc test. a: *p* < 0.050, b: *p* < 0.010, c: *p* < 0.001 vs. controls; *: *p* < 0.050, ***#***: *p* < 0.010, $: *p* < 0.001 last time point vs. intermediate time point.

Spatiotemporal fibrotic changes were investigated by calculating the evolution of the relative proportion of compact and scattered fibers in each ROI (Table 5) and of their abundance in the tissue (Figure 10).

In livers, the compact fibers mostly located in the perivascular connective tissue and the bridges, while scattered fibers were predominant in the parenchyma, irrespectively to the fibrotic status (Table 5). The modest increase in collagen amount detected in the perivascular area of CCl_4_-treated livers was attributed to a local enrichment in compact fibers (Figure 10A). The bridges showed gradual increase in the abundance of both fiber types upon CCl_4_ treatment, with compact fibers being twice predominant over the scattered fibers (Figure 10B). Collagen fibers that accumulated in the parenchyma after 7 weeks of treatment displayed mostly a scattered phenotype (Figure 10C).

In normal lungs, all compartments comprised both compact and scattered fibers. Upon fibrosis induction, higher proportion of scattered fibers was detected around the air ducts (both dosing regimen) and the vessels (BLM-acute only) (Table 5). In the parenchyma, bleomycin did not change the fiber proportion (two-thirds scattered, one-third compact) (Table 5), presumably because both fiber types accumulated in this ROI (Figure 10F). The two regimens increased the abundance of scattered fibers around vessels whereas a net increase in compact fibers was detected only in the chronic model (Figure 10D). The area surrounding air ducts remained essentially unaffected (Figure 10E).

In normal kidneys, the inner and outer medullas were mostly made up of scattered fibers, while the cortex was enriched in compact fibers. Cisplatin treatment did not change these proportions in the medulla but reversed the balance in the cortex (Table 5). Looking at the regional abundance per fiber type of fiber, cisplatin increased collagen deposits in all compartments, especially in the form of scattered fibers. Compact fibers also accumulated in all ROIs of cisplatin-treated kidneys compared to controls, particularly in the cortex in a time-dependent manner (Figure 10G–I), pointing to the cortex as a primary site of dense interstitial fibrosis.

## 4. Discussion

Whole slide imaging and modern DIA at high resolution enlarge the possibilities for accurate quantification. In the present study, we exploited DIA to address the said limitations of analyzing PSR stained sections. We studied three organs in which assessment of fibrosis is of clinical relevance. For this, we developed algorithms to (1) focus the analysis on parenchymal compartments and exclude physiologically collagen-rich structures and (2) quantitatively describe the morphological organization of PSR-stained collagen fibers, here defined as compact or scattered.

### 4.1. Novelty, Advantages, and Limitations of the Proposed Method

In line with previous comparisons of red-component (RGB-G) vs. color-specific threshold for PSR staining analysis [22], we found the RGB-based method relatively poorly sensitive, and particularly for detecting early or faint fibrosis. The PSR_OPT_ algorithm was thus optimized to be more sensitive and more specific to PSR staining, and minimally dependent on the thresholding. Similar to [20] on severe cardiac fibrosis, our method exploited the red-green contrast filter for PSR detection. However, thanks to the addition of other preprocess filters, the dynamic range of detection of PSR_OPT_ was not only (i) more robust to naturally occurring intra- and inter-studies variations, but also (ii) ampler than that of PSR_RGB_ method (illustrated in the Supplementary Material 2). Indeed, the use of hue-saturation-value (from which derives chromaticity filtering) can explain the gain in robustness to staining variations and optimal detection of faint signal [41].

The analysis of the entire tissue (*in toto* results) is a fast, simple and reliable tool for global detection of accumulated collagen. However, thanks to the regionalized quantifications, we can reveal additional changes, unsuspected or hidden in the global analysis. We provide thereby a tool to (i) evaluate whether fibrosis occurs in a regionalized manner or not, (ii) quantitatively document the relative distribution of collagen according to the histological structure, and (iii) quantify local collagen accumulation in the specific ROI(s). It is thus now possible to finely characterize fibrosis specificities in various organs using the same methodology (as discussed below).

Finally, our method offers a morphometric analysis of the organization of collagen deposits. Beside collagen content, automated fiber segmentation enables to distinguish small intertwined fibers from large clumps, each collagen segment being classified according to the mean intensity value of all the pixels that compose it. Thus, the segmentation in ROIs and the classification in fiber types offer a refined description of fibrosis for assessing spatiotemporal changes in a finely tuned manner [21].

Our method has some limitations:

(1) Intrinsically, the analysis of tissue section is an incomplete representation of the three-dimensional structures.
(2) Despite the computer-assisted recognition of the structures, the validation of the ROIs remains essential. This manual correction is a non-negligible source of error.
(3) The outcomes are defined by an arbitrary-defined threshold, based on visual evaluation. The developed PSR_OPT_ algorithm limits the impact of adjusting threshold. Robustly standardized histological procedures are still essential before quantitative DIA.
(4) The classification into compact and scattered fibers was not based on the collagen nature or effective physical parameters but on the rationale estimation that denser or thicker collagen fibrils contain more basic residues binding the dye and are therefore more intensively stained.

### 4.2. Biological Results

#### 4.2.1. Liver Fibrosis

First, the progressive 2- and 3-fold accumulation of collagen deposits found after 2 weeks or 7 weeks exposure to CCl_4_ was in line with existing literature [42,43]. In accordance with previous analyses in the same model [44,45], our regionalized analysis located most of the accumulated collagen in the bridges. This local enrichment of collagen in the bridges could be attributed to both compact (predominant) and scattered fibers. This agrees with earlier reports showing a relative increase in aggregated fibers with extended exposure to CCl_4_ [44]. Bridges have been shown to be rich in type I, III and IV collagen [42,45], with a prominent role of type III and IV during fibrosis progression [19,45]. Since PSR indifferently stains these three types of collagens [5,6,46], our results should reflect any change in the sum of these different fibers. Besides bridges, we also evidenced larger amount of collagen deposits in the parenchyma after long-term treatment (7 weeks), potentially corresponding to small or early forming deposits. Usually not integrated in the pathological descriptors, this information obtained by regionalized analysis however indicates a worsening of the disease. Together, our data confirm the progressive local accumulation of collagen in the bridges in response to CCl_4_ in a time-dependent manner, starting as scattered fibers and evolving to a more compact phenotype. Whether the distribution of scattered vs. compact fibers in the bridges and parenchyma reflects the enrichment in one type of collagen and/or reflects disease progression or resolution remains to be elucidated. 

We can conclude that CCl_4_ hepatotoxicity is characterized by the accumulation of dense collagen fibers around vessels, and a strong induction of the bridging fibrosis that progresses with exposure time.

#### 4.2.2. Lung Fibrosis

Bleomycin, irrespectively of the instillation protocol, induced a significant increase in collagen. The histological aspect of bleomycin-treated lungs fairly recapitulates the observations of the fundamental study by Degryse and Lawson [36]. The authors noted patchy areas of parenchymal fibrosis in the acute model, while the chronic model was characterized by coexistence of normal lung, dense scar, active remodeling with interstitial collagen deposition, and alveolar hyperplasia. In our specimens, we saw no dense scar, while we noted foci located close to vessels in a variable proportion between samples. The regionalized analysis pointed to a predominant parenchymal fibrosis in bleomycin-treated lungs, irrespective of the administration protocol but with a greater extend in the repetitive model. This interstitial fibrosis was a mixture of compact and loose fibers, with an increasing role of the compact fibers in the chronic model, suggesting a more severe status after repeated instillation [47]. Lung fibrotic regions have been shown to be rich in type IV collagen [48], which does not organize in dense beams of fibers, possibly explaining the predominance of scattered fibers in our study. Our results also suggest a local effect in the direct vicinity of blood vessels. First, we noted an accumulation of fibro-inflammatory foci close to the vasculature. Second, the two-classes segmentation of fibers showed a perivascular enrichment in scattered fibers in all treated lungs and an accumulation of dense fibers in this region only in chronic-treated lungs. This suggests that fibrosis induced by chronic exposure to bleomycin is more compact, perhaps more mature, and/or less subject to resorption than after a unique instillation [36]. Our results may support the concept of vascular alterations in the initiation of interstitial fibrosis and disease progression, as already proposed [49,50]. Interestingly, the formation of vessel-related structures seen by medical tomography in fibrotic patients can predict the likelihood of decline of the forced vital capacity [51] underlying the key role of vasculature in the disease. Bleomycin-induced fibrosis causes bronchial dilation [52], a phenomenon also seen in patients. In our study, while the collagen amount at the margin of air ducts remained unaffected in fibrotic samples, an increase proportion of scattered fibers was noted in this ROI. It is possible that fibers are mechanically stretched, and therefore appear less dense.

In conclusion, bleomycin induces a parenchymal lung fibrosis, and our data support a pathological action on the vasculature.

#### 4.2.3. Kidney Fibrosis

In patients, chronic use of cisplatin causes significant tubular atrophy and interstitial fibrosis with relative glomerular sparing. The repeated low-dose model used here mimics this condition more closely than the standard models (with unique and higher dose) in terms of effect on kidney function, kidney damage (interstitial cortical fibrosis) and survival rate [38].

The samples used here recapitulated the 3-fold increase in collagen deposition after 4 weeks of cisplatin as documented [38].

However, our sensitive method allowed to detect fibrosis as early as 3 weeks after the initiation of the cisplatin treatment. At this time-point, collagen accumulated in the cortex, essentially in the form of scattered fibers. One week later as fibrosis progressed, fibers were more compact, with no impact on large cortical vessels. These observations support the current understanding of cortical uptake and tubular alteration by cisplatin leading to fibrosis in these regions, despite vasculature injury [37]. Both the inner and outer medulla displayed collagen accumulation, in line with recent untargeted metabolomics data showing higher sensitivity of the medulla to cisplatin than the cortex [53]. This increase was characterized by a strong accumulation of scattered fibers, but also of compact fibers, suggesting fibrosis maturation similarly to the cortex.

### 4.3. Perspectives for Digital Pathology and Research

We demonstrate here the relevance and reproducibility of analyzing the local collagen accumulation in specific tissue compartments, *a fortiori* in the form of compact or scattered fibers with accessible material (paraffin sections), using a standard method (PSR staining) and a standard imaging system (brightfield slide scanner). Nevertheless, to go further in this characterization, complementary techniques are needed.

First, as illustrated in this work, an unbiased definition of the structures of interest for the different organs is needed, especially owing the possibility to fully automatize this key step by artificial intelligence [17,54,55].

Second, PSR stained sections can then be visualized with brightfield microscope but also under circular polarized light (although polarization data remain matter to debate for their interpretation) or fluorescent illumination, the latest possibly being as sensitive as the second harmonic generation for collagen detection [56,57,58]. Therefore, the validation of our morphometric classification of the compact vs. scattered fibers may be addressed by one of these relatively accessible methods in the future. Alternatively, label-free second-harmonic generation/two-photon excited fluorescence microscopy may address this question. Indeed, when combined with deep learning, this is used to reveal the spatiotemporal changes in liver fibrosis [27,31,32,44,59,60]. This technology is promising since the recent marketing of a slide scanner using this microscopy method for clinical applications [61]. Furthermore, another type of advance microscopy (staining-free fluorescence lifetime imaging) is proposed to distinguish type I vs. type III collagen [62], but this claim still needs validation by complementary approaches such as immunohistology. With such technologies, additional morphometric features can be investigated such as the fiber length, diameter, orientation, alignment, anisotropy, or shape factors (i.e., waviness) which have been associated with pathologies, developing the concept of pathology-associated collagen signatures [63,64]. Such classification could ultimately serve to stratify the degree of fiber density and compactness using conventional microscopy.

## 5. Conclusions

The golden rules for a reliable method are simplicity (easy to handle, low manpower need, etc.) and reproducibility within and between studies. Our new digital method to detect collagen fibers using PSR staining in brightfield is simple, robust, efficient, and tissue-transposable, allowing inter-studies and multi-organ comparison. The regionalized analysis offers a spatiotemporal characterization of collagen deposit organization and the proposed method of fiber classification enables pattern recognition to better describe fibrosis characteristics. Beside the useful place of PSR for accurate diagnosis, the present work implicates that the analysis opportunities offered by this staining are often underexploited.

## Figures and Tables

**Table 1:** Characteristics of regions-of-interest (ROIs) and regions-of-exclusion (ROEs).

	Total Tissue Area (mm^2^)	Regions-of-Interest (ROIs)				Regions-of-Exclusion (ROEs)
	Mean (±SD)					
			**Relative Area (%)**		***p*-Val**	
			**Mean (±SD)**			
			**ControlOrgans** **(n = 5)**	**Fibrotic Organs ^1^** **(n = 5)**		
**Liver**	43.3 (13.9)	Perivascular tissue	1.20 (0.32)	1.92 (0.70)	0.007	Capsule
		Bridges	0.04 (0.02)	2.79 (0.30)	<0.001	Large lumens
		Parenchyma	98.76 (0.30)	95.29 (0.84)	<0.001	
**Lung**	32.77 (6.18)	Peri-air ducts tissue	4.22 (0.39)	4.24 (0.81)	0.950	Pleura
		Perivascular tissue	2.94 (0.78)	2.93 (0.45)	0.992	Dense peri-air duct connective tissue (variable) ^2^
		Parenchyma	92.85 (0.95)	92.83 (1.01)	0.973	Large lumens & arteries
**Kidney**	17.63 (1.73)	Inner medulla	4.46 (1.15)	6.49 (3.07)	0.204	Capsule
		Outer medulla	10.88 (2.32)	13.03 (2.61)	0.205	Hilum
		Cortex	84.66 (1.78)	80.48 (2.81)	0.022	Large lumens

^1^ Last time point of treatment (liver: 7w CCl_4_; lung: BLM-chronic; kidney: 4w CIS). ^2^ Strongly stained fibers surrounding very large air ducts were included in the total tissue area but not considered as a ROI. This region represented 5.02% (1.42) and 6.46% (2.12) of the total tissue area (*p* > 0.05).

**Table 2:** Liver collagen proportion per ROI. Mean percentages (±SD).

Collagen Proportion (%)	Controls	2w CCl_4_	*p*-Val	7w CCl_4_	*p*-Val
Perivascular	85.71 (11.10)	64.22 (7.71)	0.006	45.06 (11.40)	<0.001
Bridges	1.89 (1.94)	32.09 (7.72)	<0.001	46.27 (8.44)	<0.001
Parenchyma	12.40 (9.24)	3.69 (1.24)	0.037	8.67 (4.04)	0.334
Total	100%	100%		100%	

**Table 3:** Lung collagen proportion per ROI. Mean percentages (±SD).

Collagen Proportion (%)	Controls	BLM-Acute	*p*-Val	BLM-Chronic	*p*-Val
Perivascular	5.46 (1.58)	4.89 (1.84)	0.556	5.10 (0.80)	0.709
Peri-air ducts	19.64 (5.45)	8.66 (2.61)	0.001	9.81 (1.43)	<0.001
Parenchyma	74.91 (6.39)	86.44 (4.33)	0.002	85.09 (1.74)	0.004
Total	100%	100%		100%	

**Table 4:** Kidney collagen proportion per ROI. Mean percentages (±SD). Cortex was sub-analyzed to distinguish the parenchyma ^(1)^ and the large cortical vessels ^(2)^.

Collagen Proportion (%)	Controls	3w CIS	*p*-Val	4w CIS	*p*-Val
Inner medulla	10.29 (7.95)	15.83 (3.78)	0.147	14.54 (4.28)	0.257
Outer medulla	4.94 (1.07)	13.06 (3.49)	<0.001	4.98 (1.62)	0.979
Cortex ^1,2^	84.78 (8.59)	71.10 (4.02)	0.004	80.48 (4.60)	0.286
Total	100%	100%		100%	
^1^ Cortex (minus vessels)	36.07 (8.00)	54.90 (4.57)	0.001	65.33 (8.51)	<0.001
^2^ Large cortical vessels	48.70 (10.52)	16.21 (7.87)	<0.001	15.15 (6.46)	<0.001

**Table 5:** Collagen proportion (%) per fiber type in specific histological compartments. Mean percentages (±SD).

			Compact/Scattered Fibers Relative Area				
	Regions-of-Interest (ROIs)	Fiber Class	Controls (n = 5)	Treatment Time-Point 1 (n = 5)	*p*-Val	Treatment Time-Point 2 (n = 5)	*p*-Val
**Liver**	Perivascular tissue	compact	81.23 (7.82)	87.41 (3.70)	0.100	86.00 (3.92)	0.194
		scattered	18.77 (7.82)	12.59 (3.70)		14.00 (3.92)	
	Bridges	compact	66.78 (9.28)	71.29 (4.61)	0.328	67.00 (6.27)	0.961
		scattered	32.22 (9.28)	28.71 (4.61)		33.00 (6.27	
	Parenchyma	compact	23.15 (3.32)	18.90 (3.55)	0.272	23.49 (8.87)	0.928
		scattered	76.85 (3.32)	81.10 (3.55)		76.51 (8.87)	
**Lung**	Peri-air ducts tissue	compact	59.61 (7.03)	41.53 (5.10)	<0.001	48.07 (5.71)	0.010
		scattered	40.39 (7.03)	58.47 (5.10)		51.93 (5.71)	
	Perivascular tissue	compact	45.53 (7.47)	36.44 (5.40)	0.026	42.81 (3.32)	0.461
		scattered	54.47 (7.47)	63.56 (5.40)		57.19 (3.32)	
	Parenchyma	compact	36.32 (6.14)	33.36 (3.64)	0.298	41.45 (2.06)	0.083
		scattered	63.68 (6.14)	66.64 (3.64)		58.55 (2.06)	
**Kidney**	Inner medulla	compact	6.04 (4.40)	7.20 (2.67)	0.696	9.95 (6.04)	0.203
		scattered	93.96 (4.40)	92.80 (2.67)		90.05 (6.04)	
	Outer medulla	compact	10.81 (7.42)	18.70 (10.41)	0.154	15.48 (6.15)	0.385
		scattered	89.19 (7.42)	81.30 (10.41)		84.52 (6.15)	
	Cortex	compact	68.47 (3.48)	39.48 (7.63)	<0.001	42.25 (6.01)	<0.001
		scattered	31.53 (3.48)	60.52 (7.63)		54.75 (6.01)	

## Supplementary Materials

The following are available online at https://www.mdpi.com/2218-273X/10/11/1585/s1. Supplementary Material 1: Computer assisted organ-specific definition of the regions-of-interest. Supplementary Material 2: Detection filters and threshold determination for the RGB-based or the PSR-optimized methods. Supplementary Material 3: RGB-based and the PSR-optimized methods used for multi-organ comparison in liver, lung, and kidney (illustration and correlation). Supplementary Material 4: Validation of the developed PSR-optimized method vs. previously described PSR-detection method on murine kidney samples.
