# Supplementary material for: Digital Image Analysis of Picrosirius Red Staining: A Robust Method for Multi-Organ Fibrosis Quantification and Characterization
Source: Biomolecules. 2020 Nov 22;10(11):1585. doi: 10.3390/biom10111585 (PMC7709042; doi:10.3390/biom10111585)

biomolecules

Supplementary Material 1: Computer assisted organ-specific definition of the regions-of-interest.

The proposed workflow is based on a first common method for tissue detection, followed by exclusion of the capsule (25 µm) or the pleura (40 µm), considered irrelevant for this study. In a second step, algorithms coding for computer-assisted definition of the ROIs and ROEs were specific for each organ. Detailed procedure is given step-by-step per organ.

1. Liver

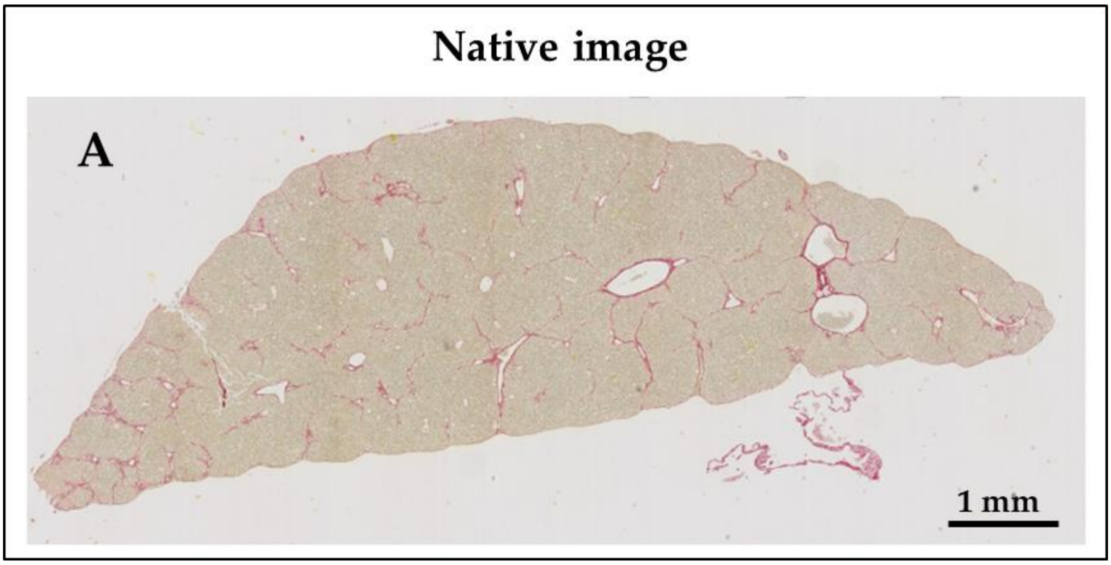

1.1. Automated tissue detection and lumen and capsule exclusion

Tissue was detected with a fixed threshold using the Contrast Red-Green filter at x10 magnification on the entire image. Holes in tissue >0.01 mm² were considered as large lumen and excluded. The capsule was excluded by tissue erosion of 25 μm (empirically defined value), and irrelevant structures and artefacts were manually discarded (**1B**).

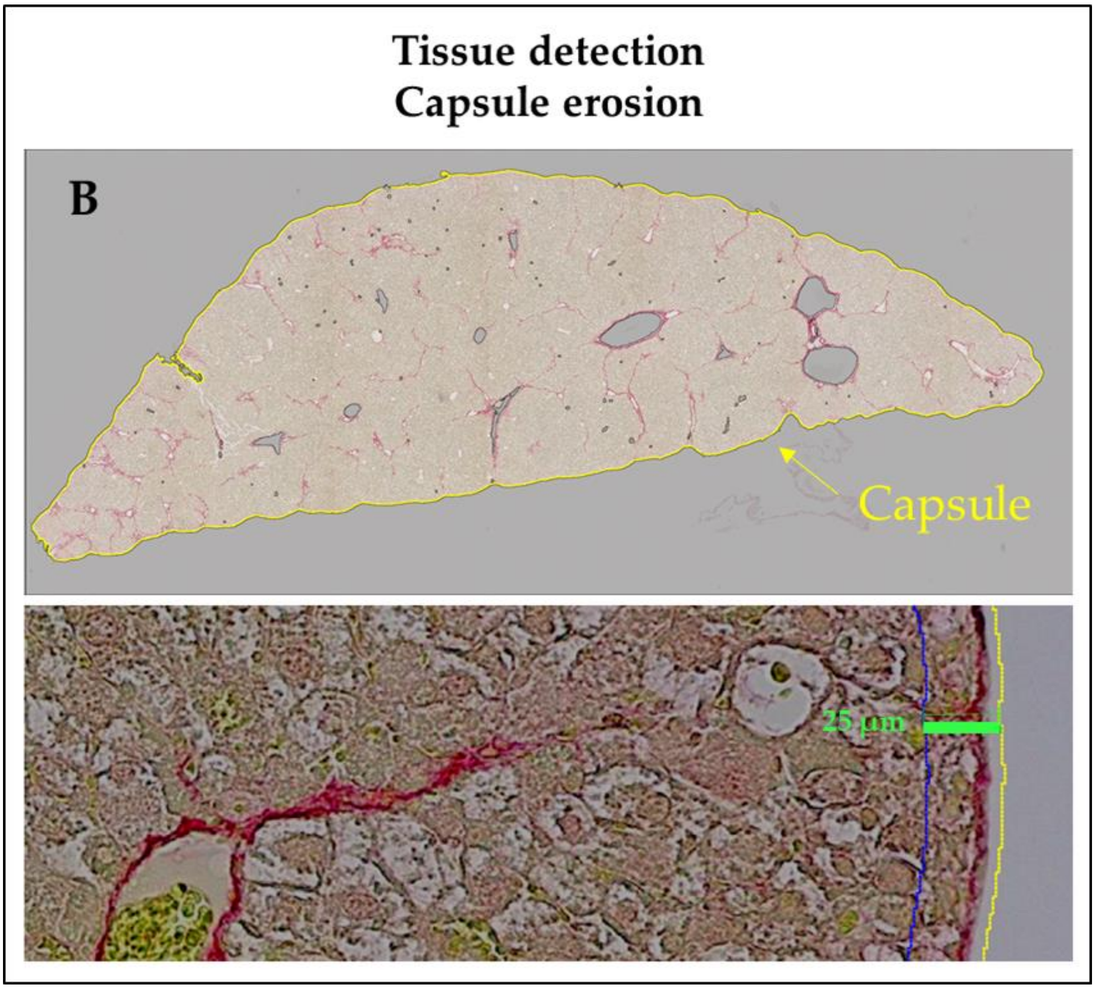

1.2. – Fully automated ROI segmentation

Within the delineated tissue, automated recognition of PSR staining was achieved at x20 magnification using a threshold classification of pixels based on a combination of RGB-G and RGB-B filtering (**C1**). By iterative dilatation and closing, positive pixels were fused (**C2**). Maximal gap between 2 segments was equivalent to 4 µm. Small segments (<100 µm²) were discarded. Then, within the delineated *tissue*, small lumen and/or lumen contiguous to blood clots (vessels) were detected using a combination of RGB-R and AEC_DAB-DAB preprocesses, with an individual area >3000 µm², and considered as *lumens* (**C3**). PSR-positive segments underwent watershed and separate objects post-processes (**C4**). Segments surrounding *lumens* were defined as *perivascular connective tissue*, whereas the others were defined as *bridges* (**C5**). The remaining tissue was considered as *parenchyma*. Manual correction was performed when required, giving a segmented image ready for analysis (**C6**).

1.2.1. Native image (**C0**)

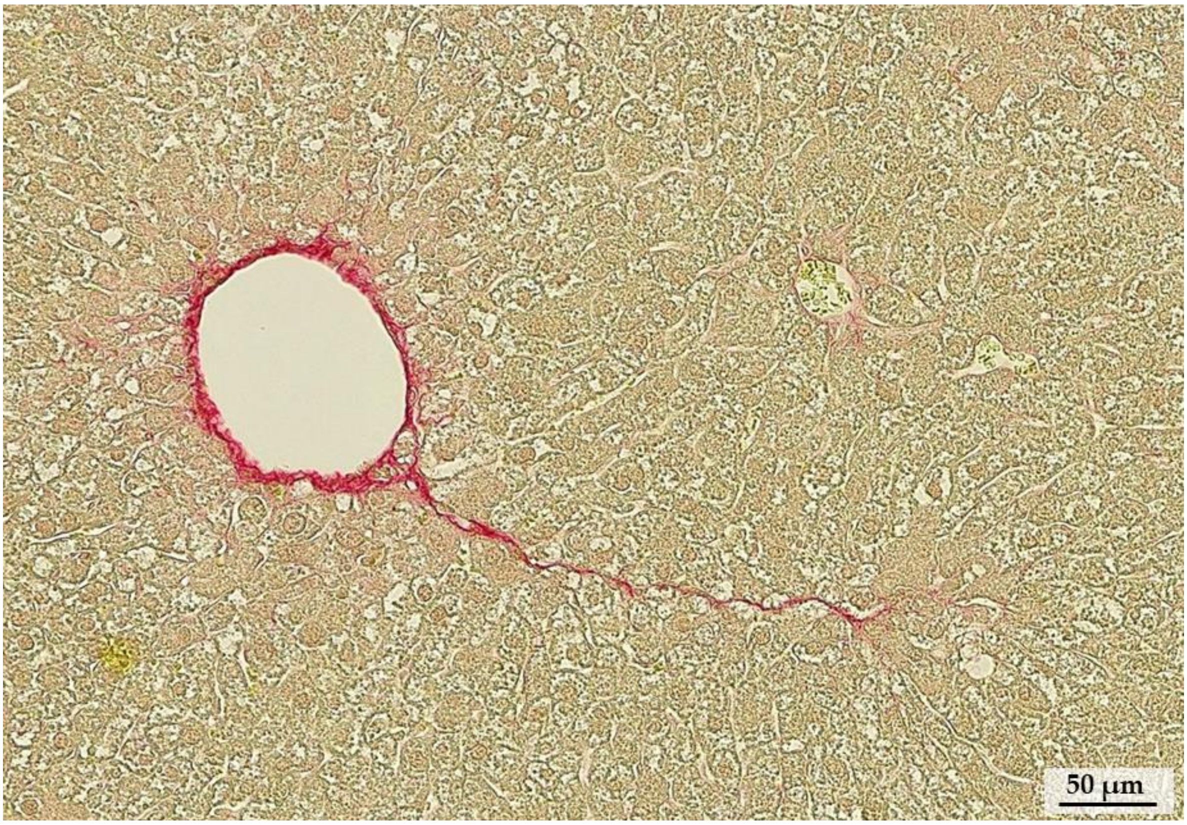

1.2.2. *Threshold (blue) to detect PSR stained pixels* (**1C1**)

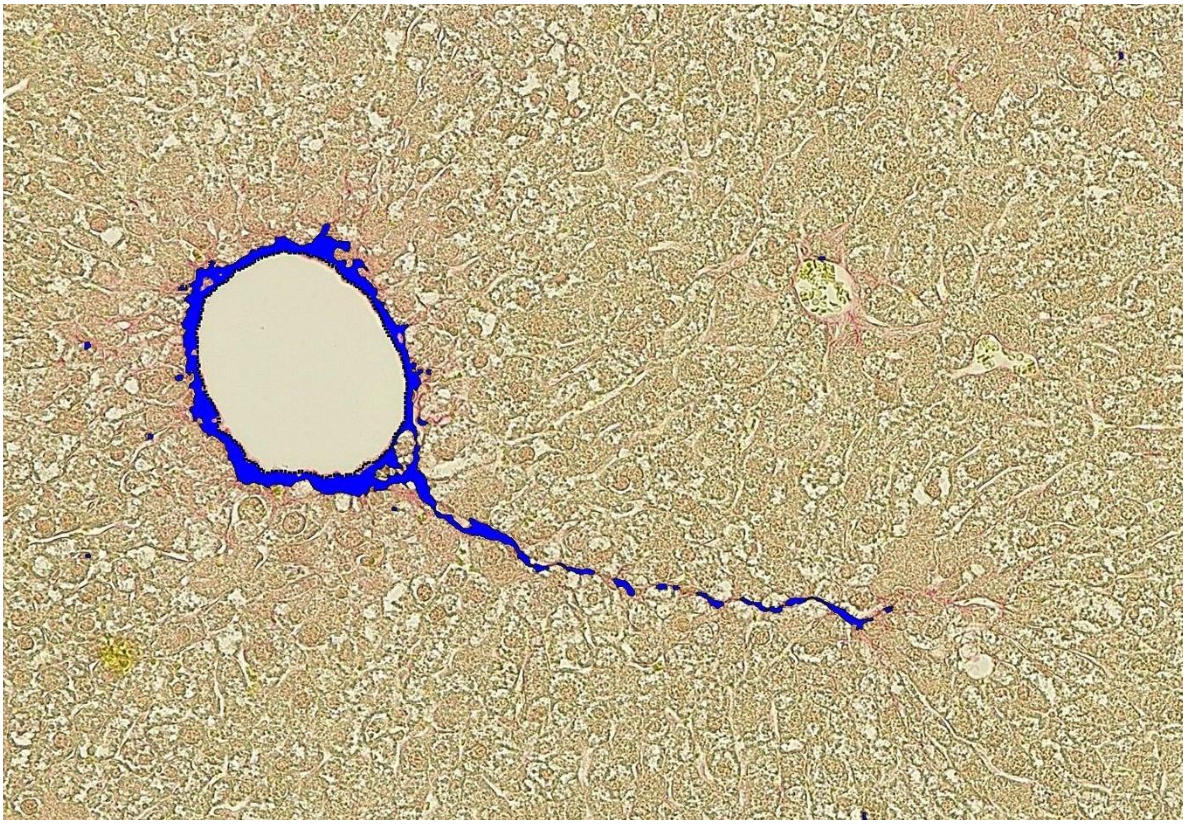

1.2.3. *Closing pixels and elimination of small pixels* (**C2**)

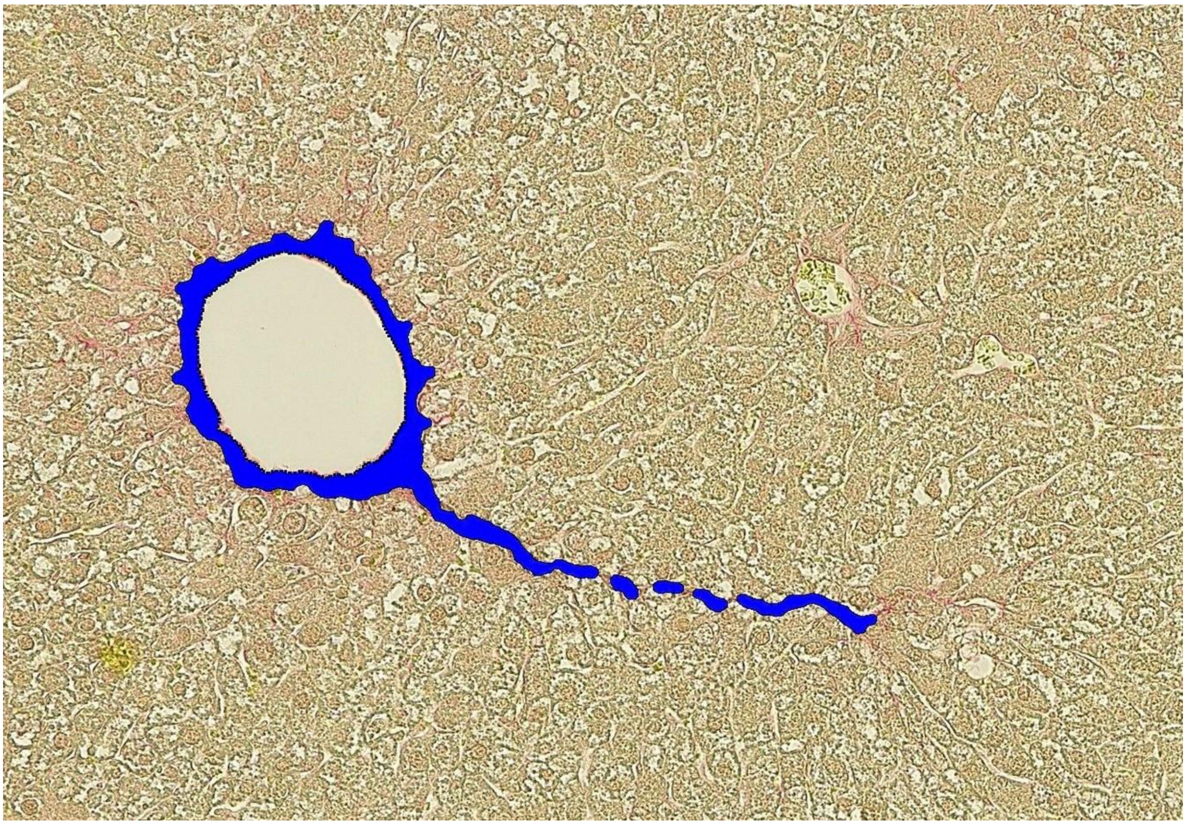

1.2.4. Lumen detection (red) (**C3**)

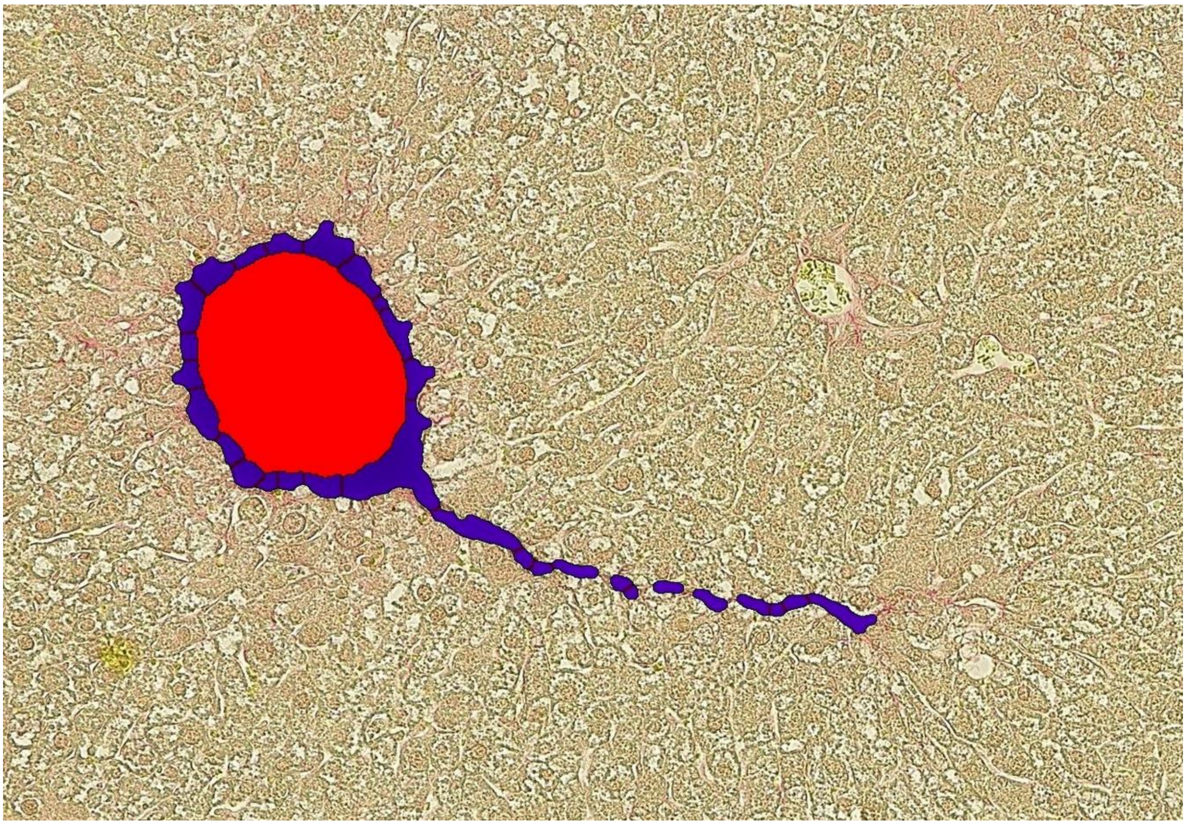

1.2.5. Watershed and separate objects, and detection of segments surrounding lumens (green) (**C4**)

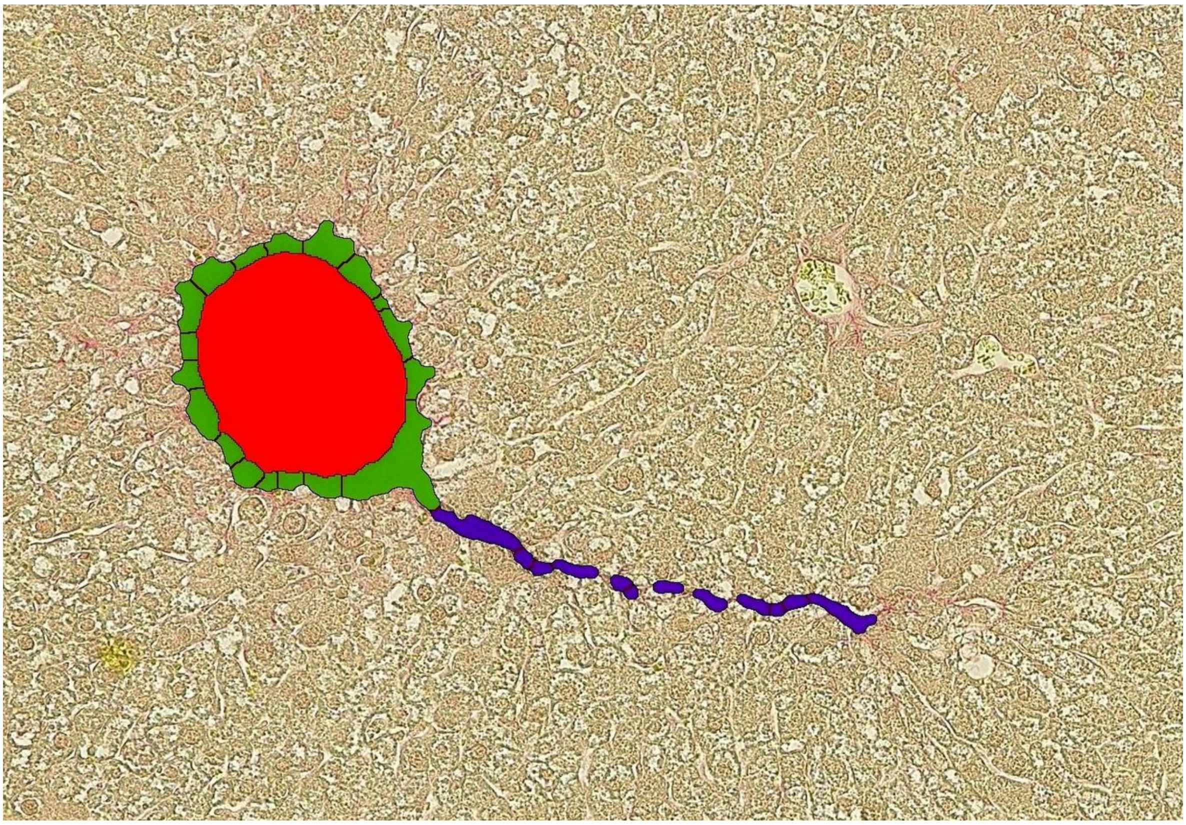

1.2.6. *Conversion in ROIs (cyan: perivascular connective tissue; red: bridges)* (**C5**)

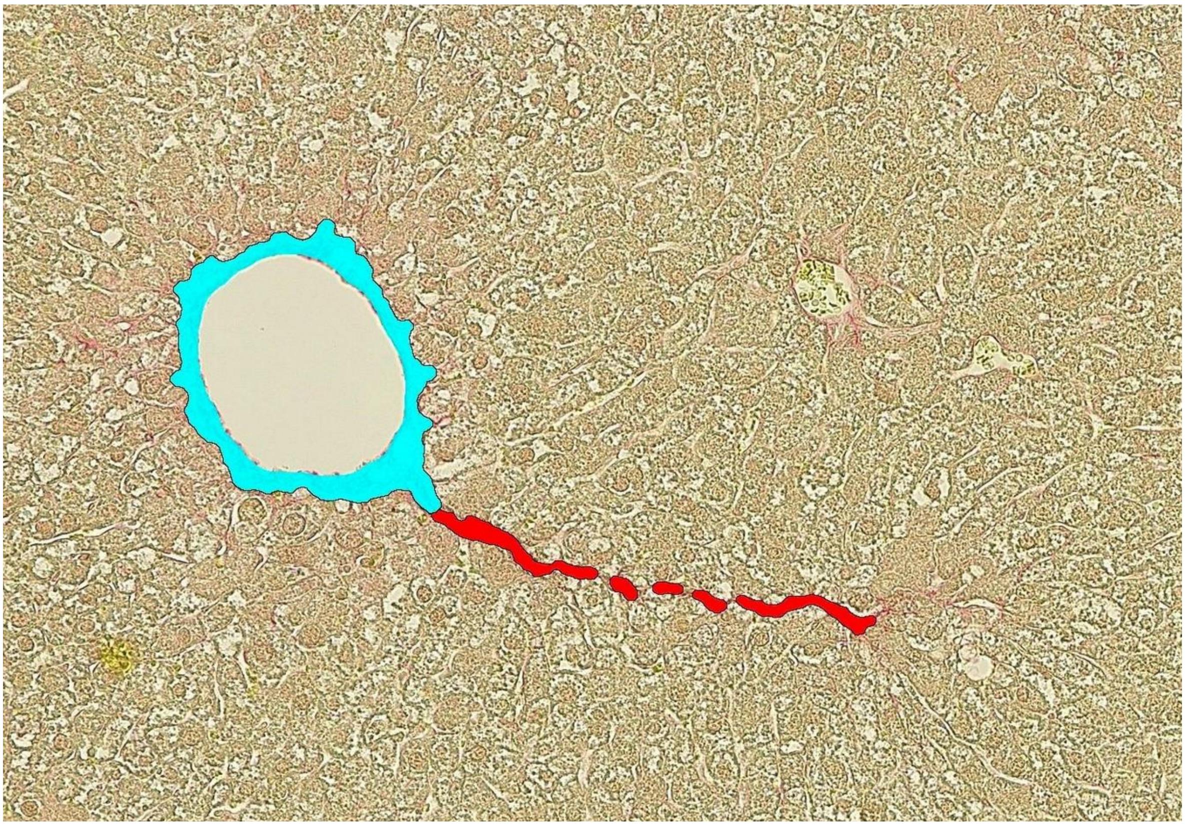

1.2.7. Segmented image of the liver (***C6***)

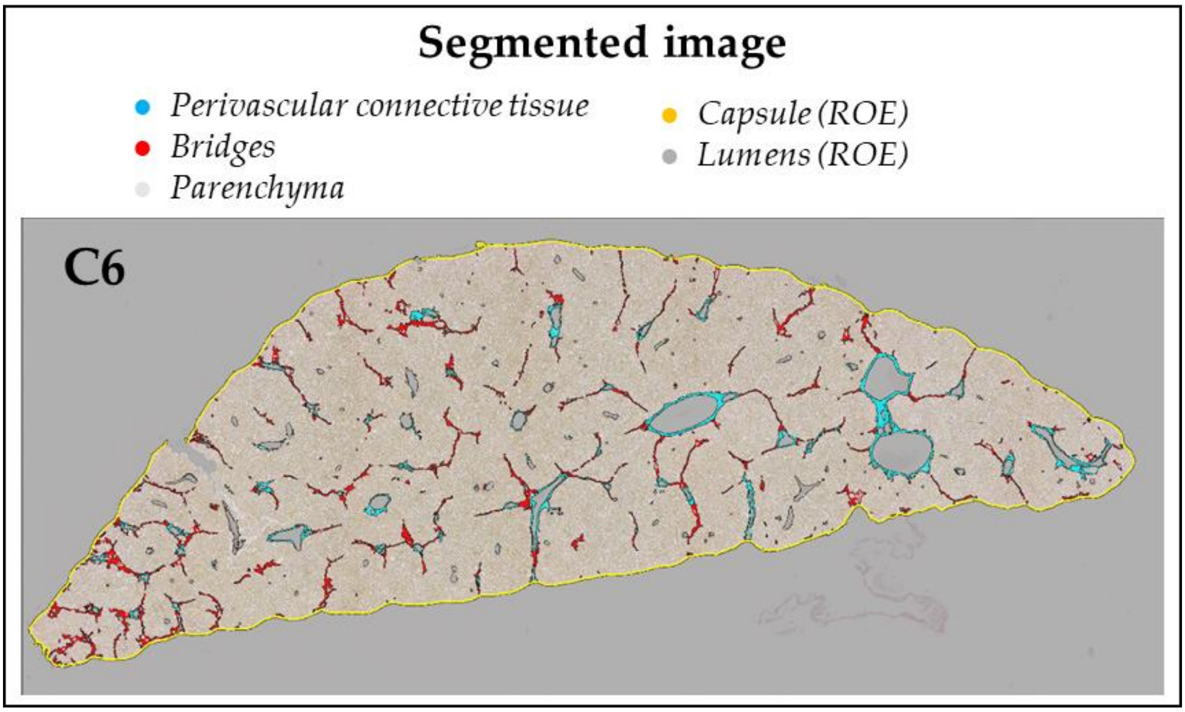

2. Lung

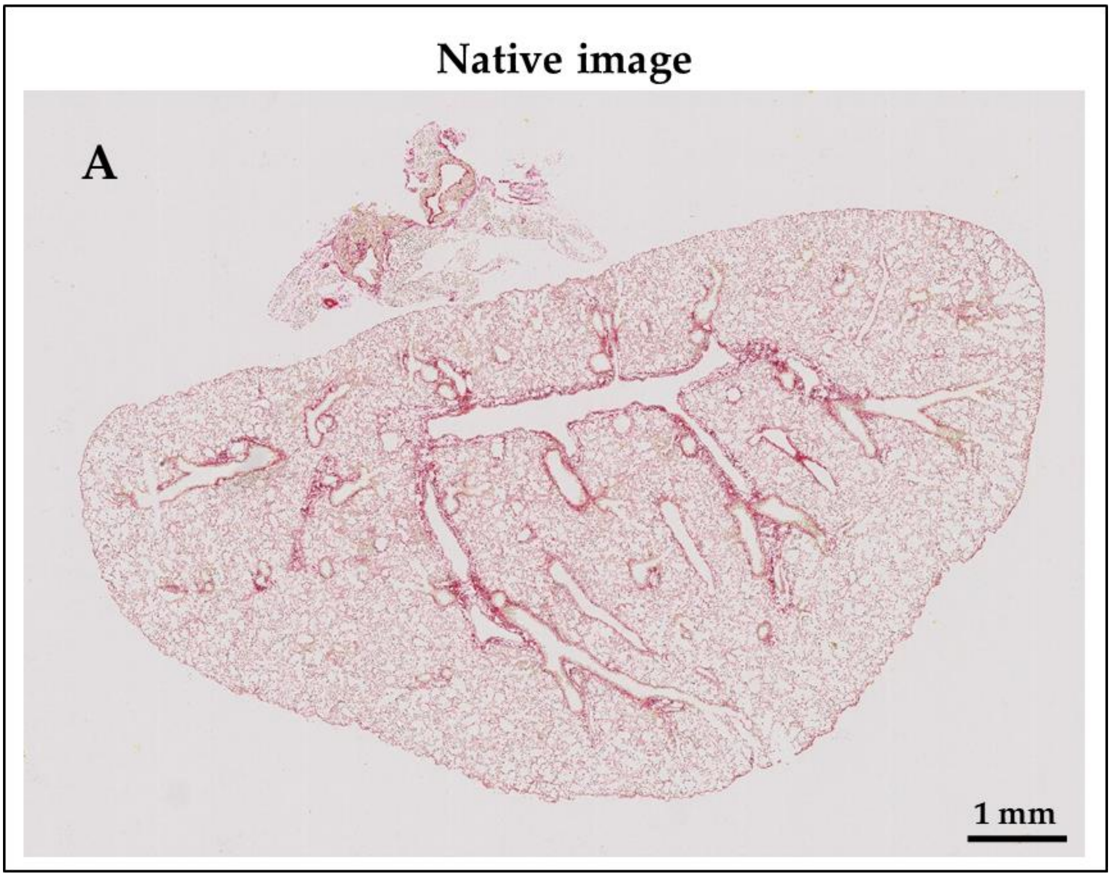

2.1. Step1 – Automated tissue detection and pleura exclusion

Tissue was detected with a fixed threshold using the Contrast Red-Green filter at x10 magnification on the entire image. The pleura was excluded by tissue erosion of 40 µm (empirically assessed, since 25 µm was not sufficient), and irrelevant structures and artefacts were manually discarded (**B**).

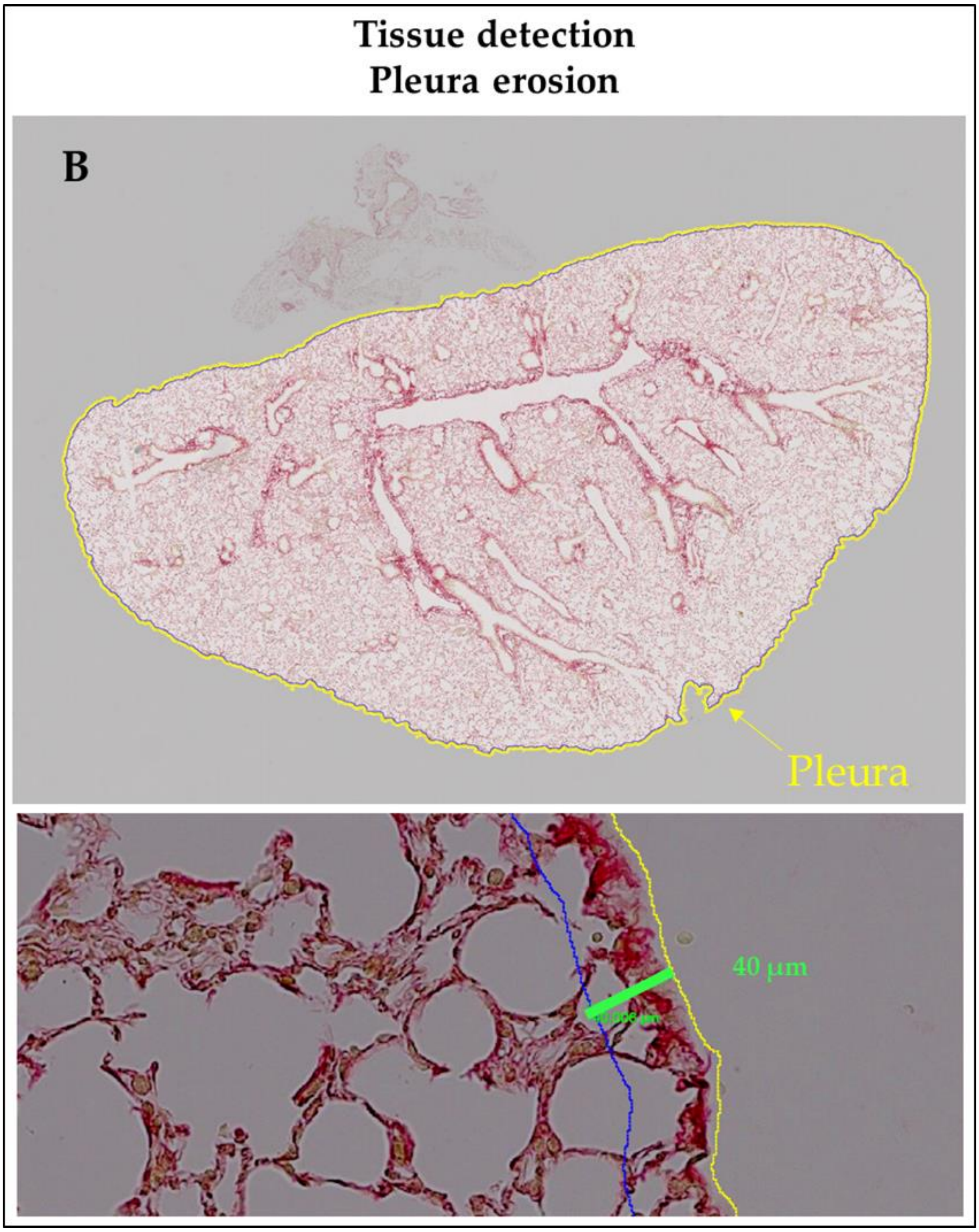

2.2. Step2 – Computer-assisted ROI segmentation

First, we observed a variable presence of large bronchi (and abundant surrounding connective tissue) on the lung sections from different mice. To avoid bias in the results interpretation, these structures were recognized by strongly PSR-stained pixels surroundings air conducting structures, and were automatically discarded (**C1**). For informative purpose, CP values were calculated and

showed that these fibers represented ~60% of total collagen, whereas the relative area of this ROI was only ~5%.

Then, series of algorithms were designed to assist ROI segmentation, at x10 magnification, but complete automation was not achievable. Within the delineated tissue, the diverse air upper conducting portions (bronchi and bronchioles) were first detected by the presence of pseudostratified columnar epithelium (stained in yellow) and the layer of smooth muscle (stained in pink). Vessels were detected as a lumen surrounded by a muscular layer (stained in pink) (**C2**). At this level, careful correction of misattributed or undetected structures (ie. structures with irregular shape) was performed manually. Once validated, a dilatation of 25 µm was performed to generate the peri air duct and perivascular regions (**C3**), giving a segmented image ready for analysis (**C4**).

2.2.1. *Heterogeneity of the peri-air duct large connective tissue* (**C1**)

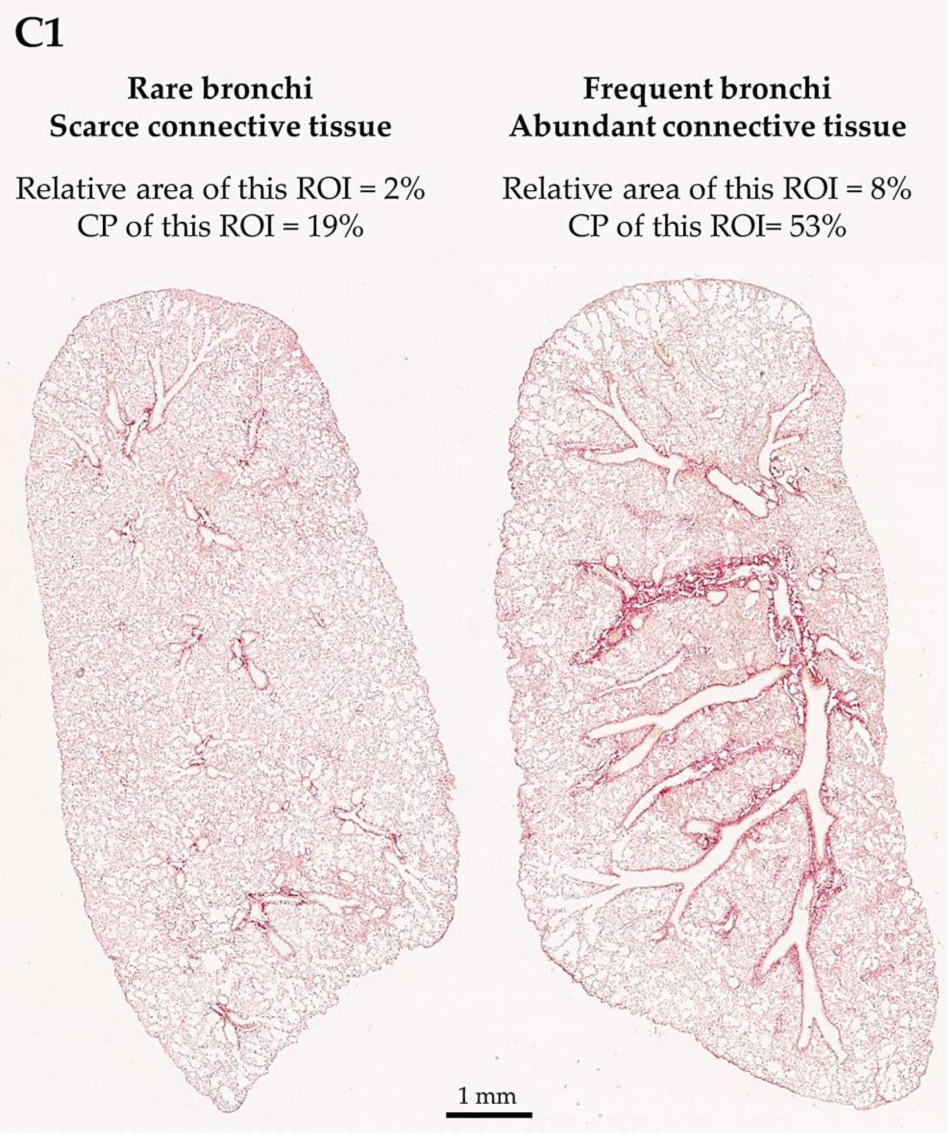

(**C1 suite**) *Native image showing peri-air duct connective tissue (ROE) surrounding a bronchiole and vessels.*

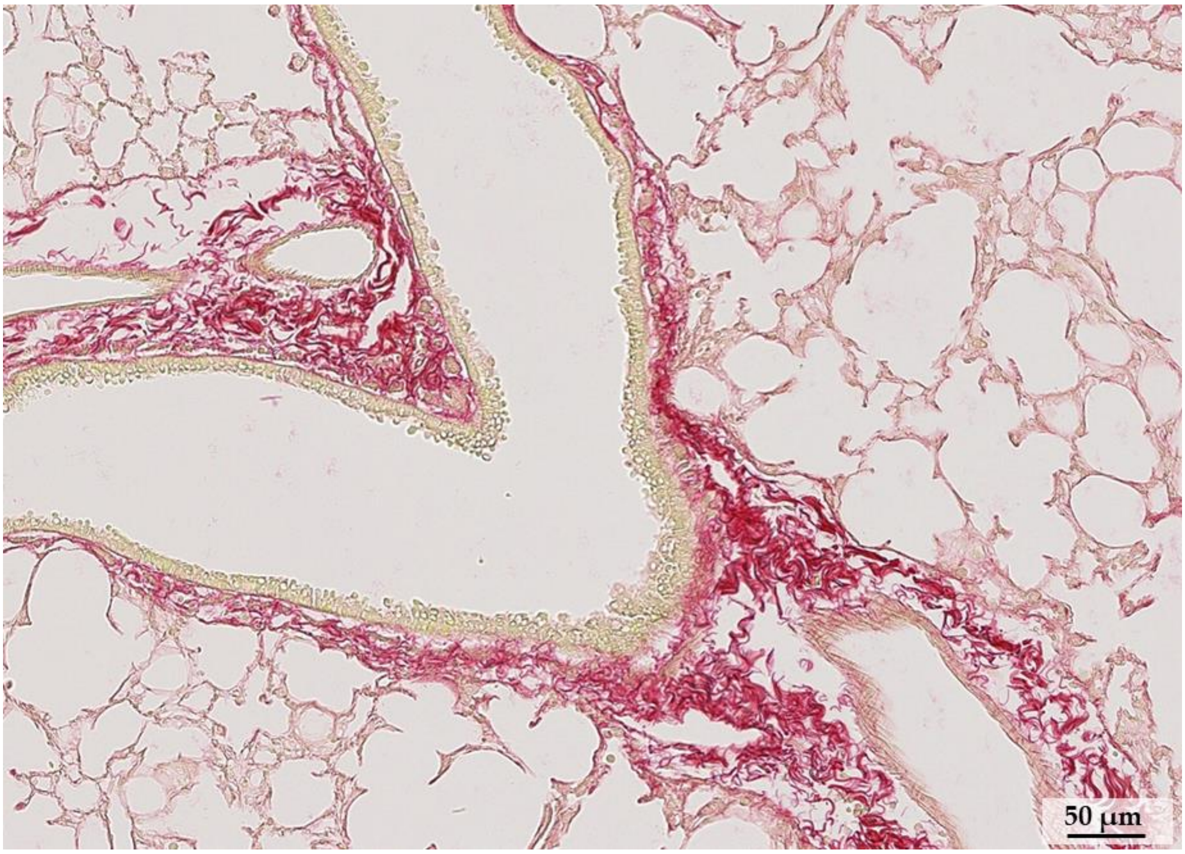

(**C1 suite**) *Exclusion of peri-air duct connective tissue.*

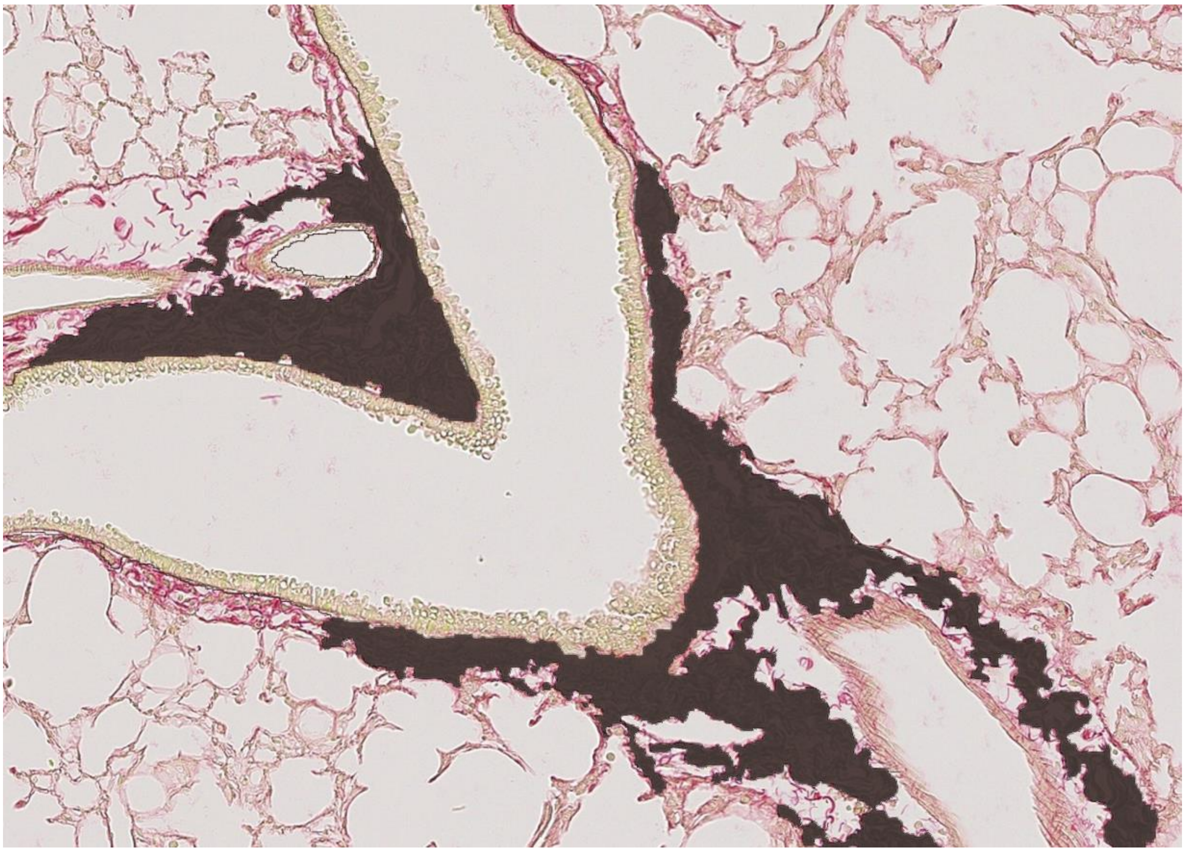

(**C2**) Semi-automated detection of upper air duct (yellow) and vessels (red), after manual validation.

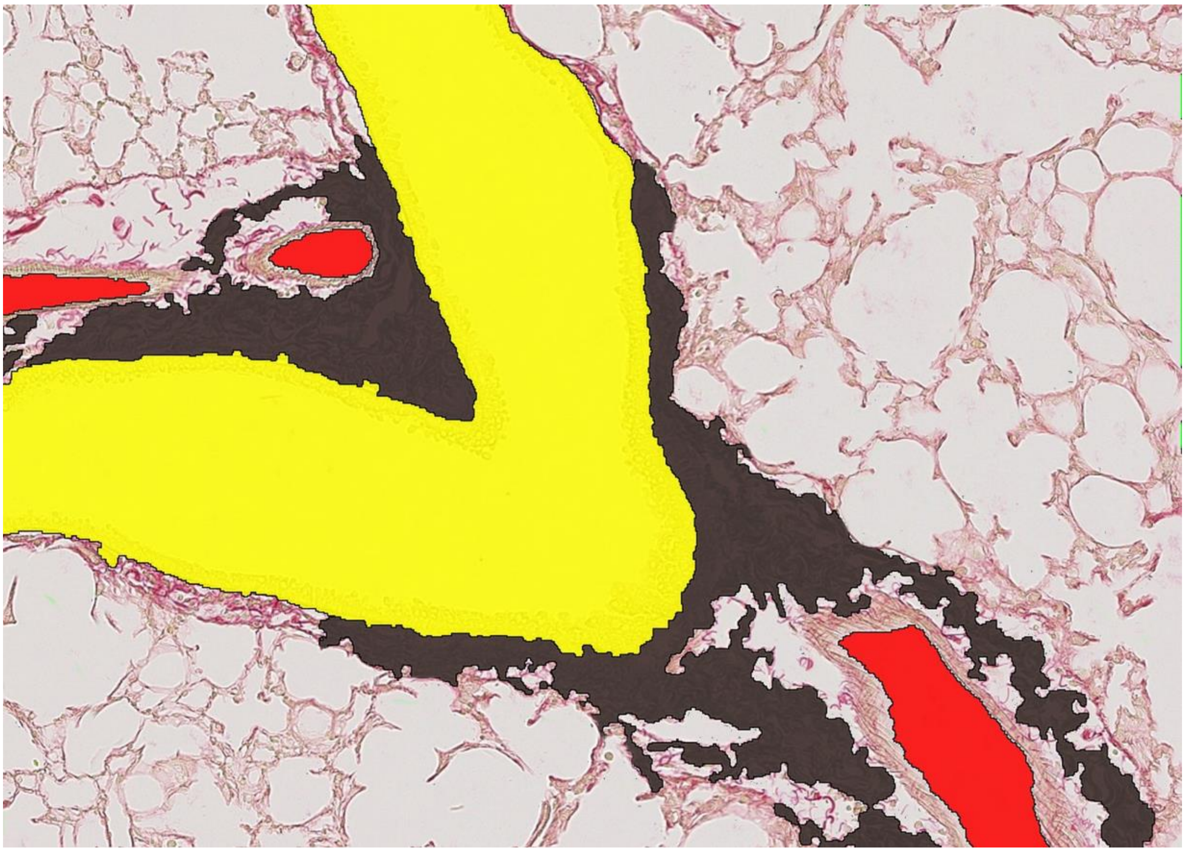

(***C3***) *Dilatation of 25 μm from upper air duct and vessels, giving the peri-air duct region (cyan) and the perivascular region (orange). The concentric regions were kept as ROI, whereas the template regions are discarded as lumens.*

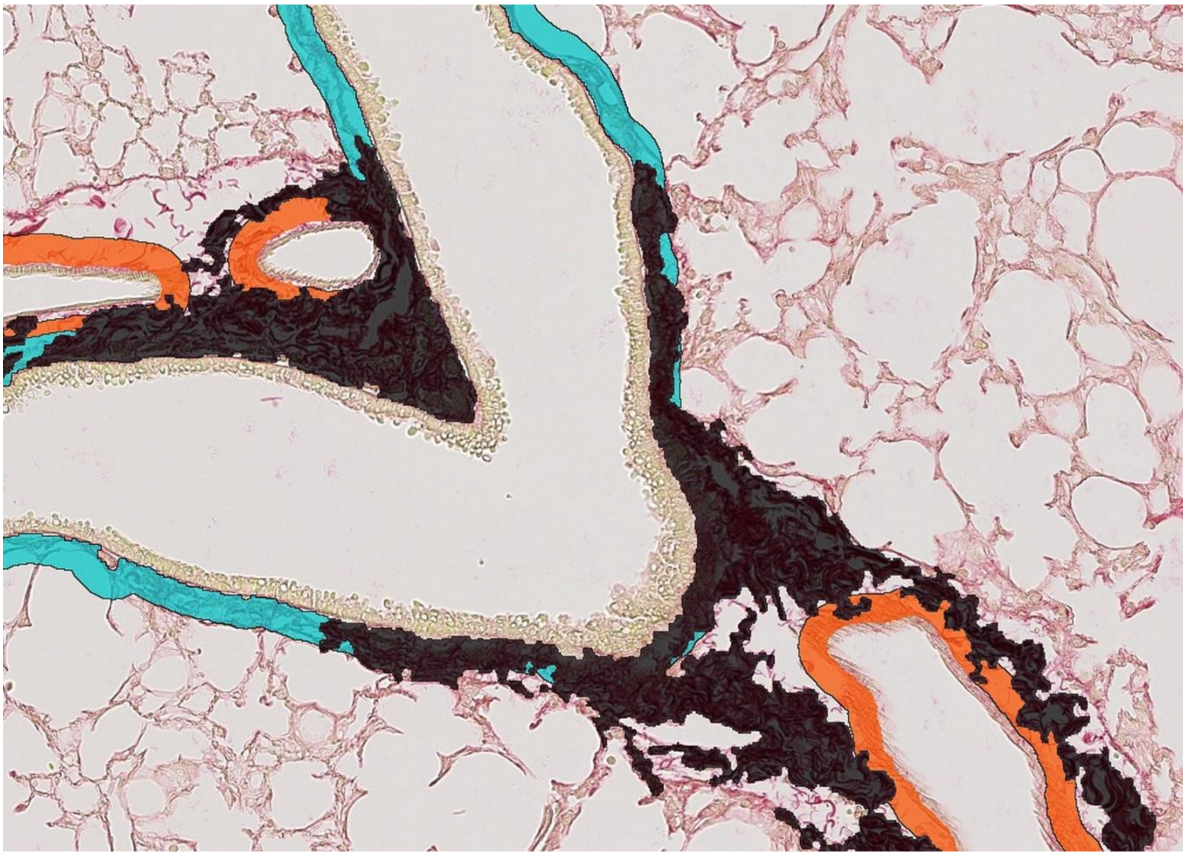

77 (**C4**) Segmented image of the lung.

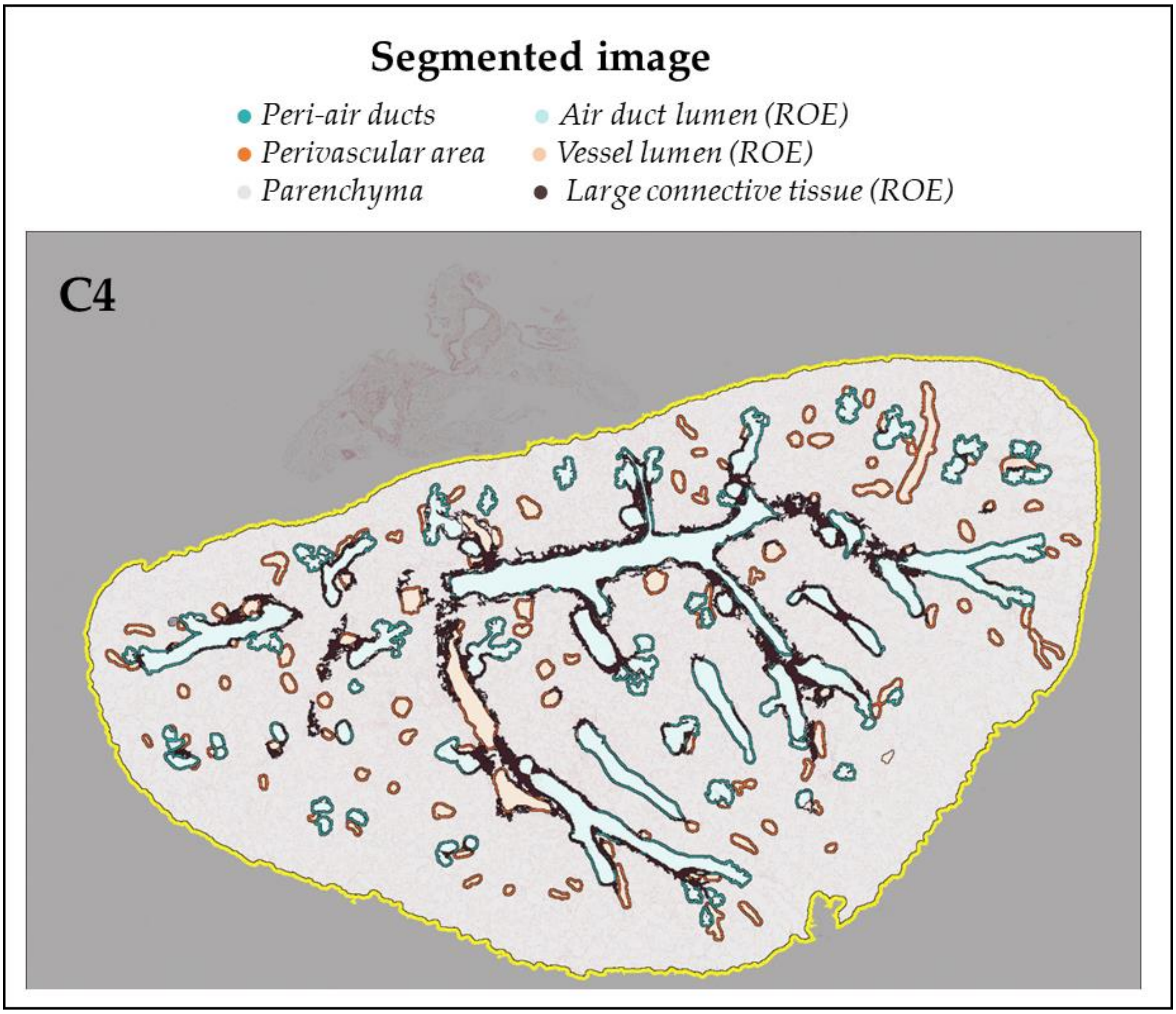

78

3. <u>Kidney</u>

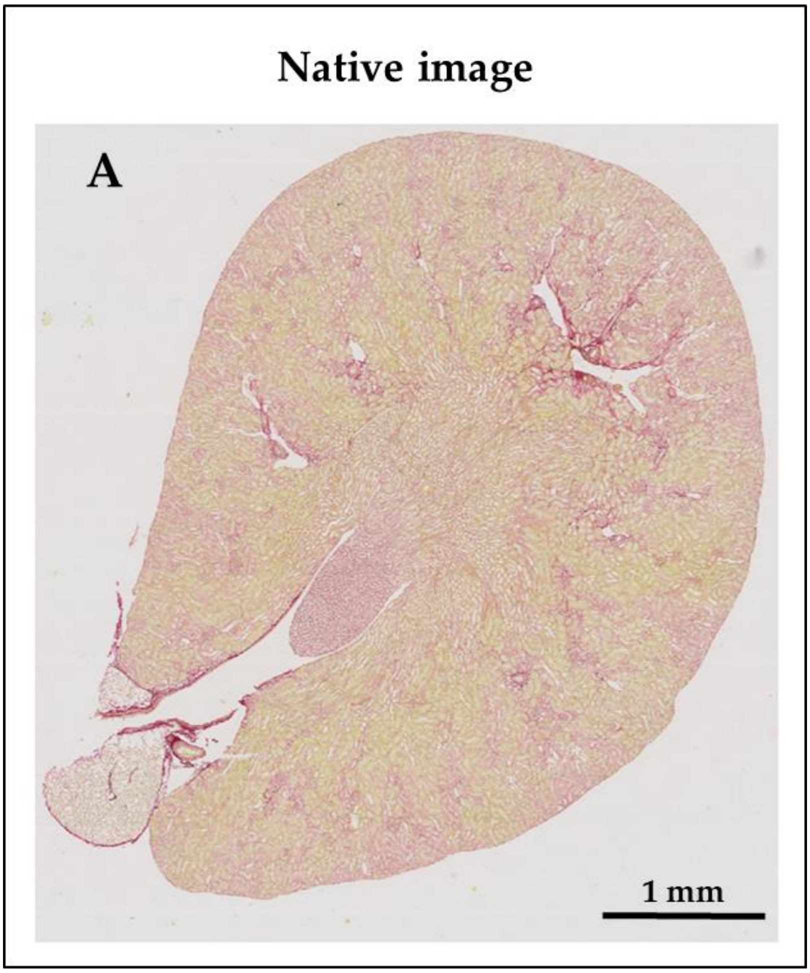

3.1. Step1 – Automated tissue detection and capsule exclusion

Tissue was detected with a fixed threshold using Contrast Red-Green filter at x10 magnification on entire image. Holes in tissue >0.01 mm² are considered as large lumen and excluded. The capsule was excluded by tissue erosion of 25 µm (empirical value), and irrelevant structures and artefacts were manually discarded (**B**).

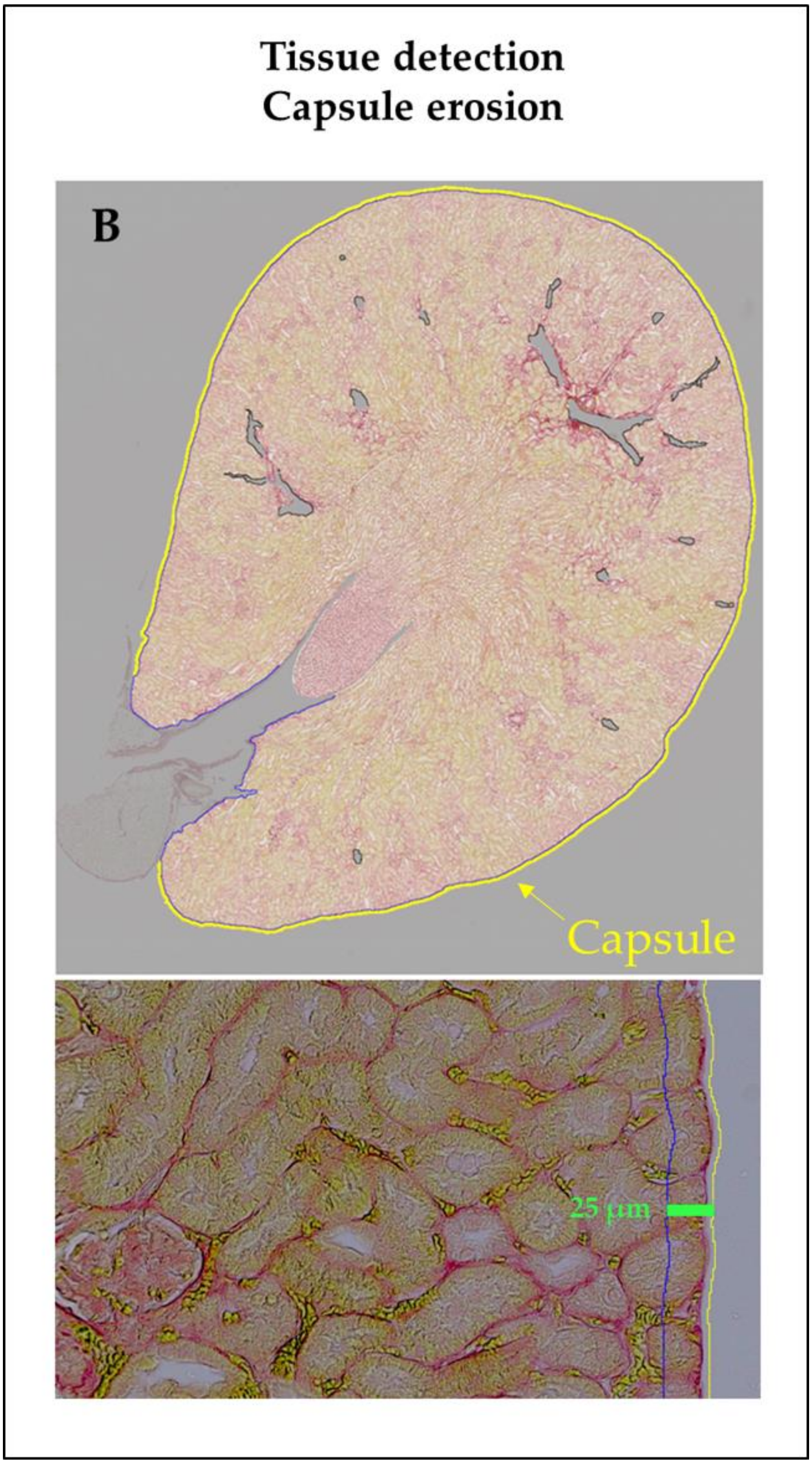

3.2. Step2 – Manual ROI segmentation

The *inner medulla*, the *outer medulla* and the *cortex* were manually delineated giving a segmented image ready for analysis (**C**). Because the phenotype observed in the cisplatin group suggested a possible staining accumulation around large vessels (<0.01 mm^2) located in the *cortex*, the *large cortical vessels* were manually delineated for a sub-analysis of the *cortex*.

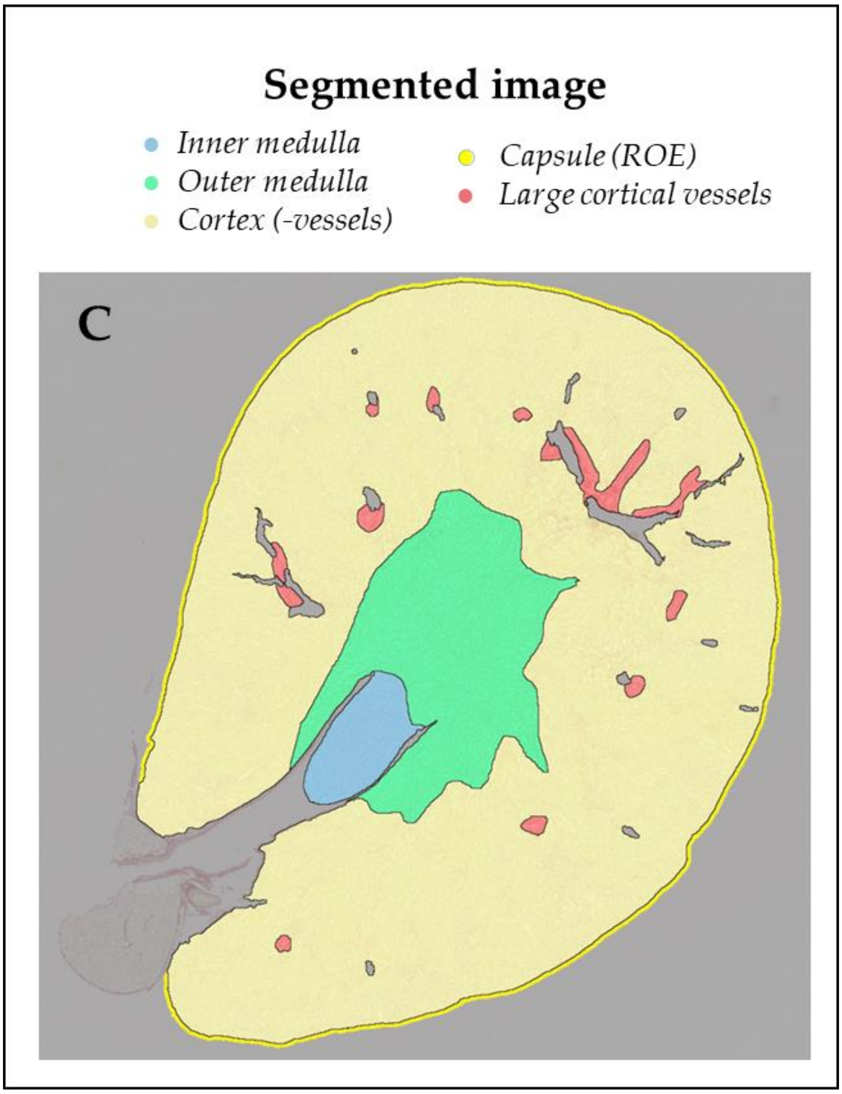

Supplement: Supplementary file 1 [file biomolecules-10-01585-s001.zip › Courtoy et al. Supplementary Material 1.pdf]
