# Supplementary material for: Digital Image Analysis of Picrosirius Red Staining: A Robust Method for Multi-Organ Fibrosis Quantification and Characterization
Source: Biomolecules. 2020 Nov 22;10(11):1585. doi: 10.3390/biom10111585 (PMC7709042; doi:10.3390/biom10111585)

Supplementary Material 2: Detection filters and threshold determination for the RGB-based or the PSR-optimized methods. An illustration is given in the liver.

Raw image

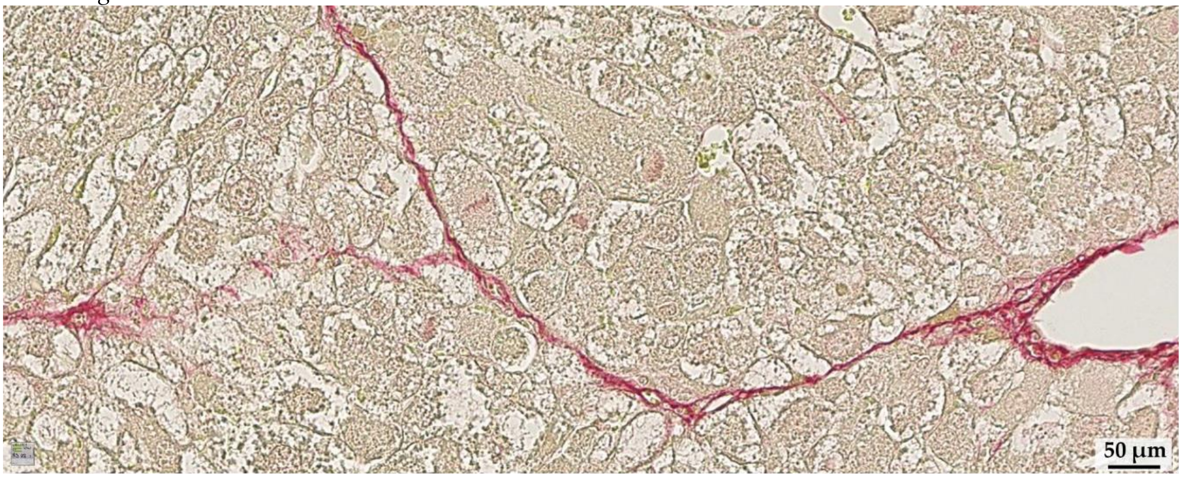

A. RGB-based method

1. Detection filter (RGB-G)

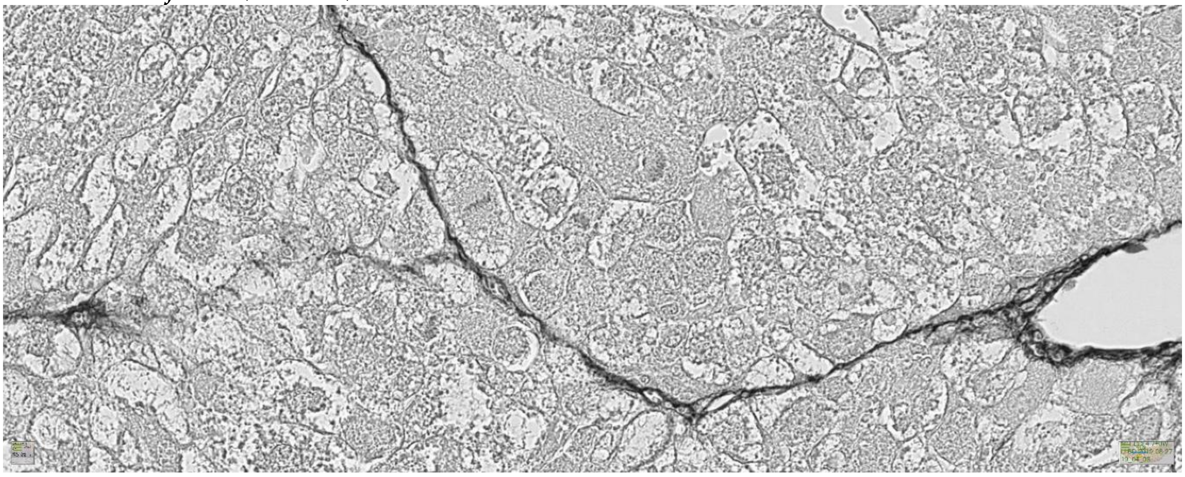

2. Result with RGB-based method

Threshold set to best fit all organs (=85). Note the presence of aspecific and undetected pixels.

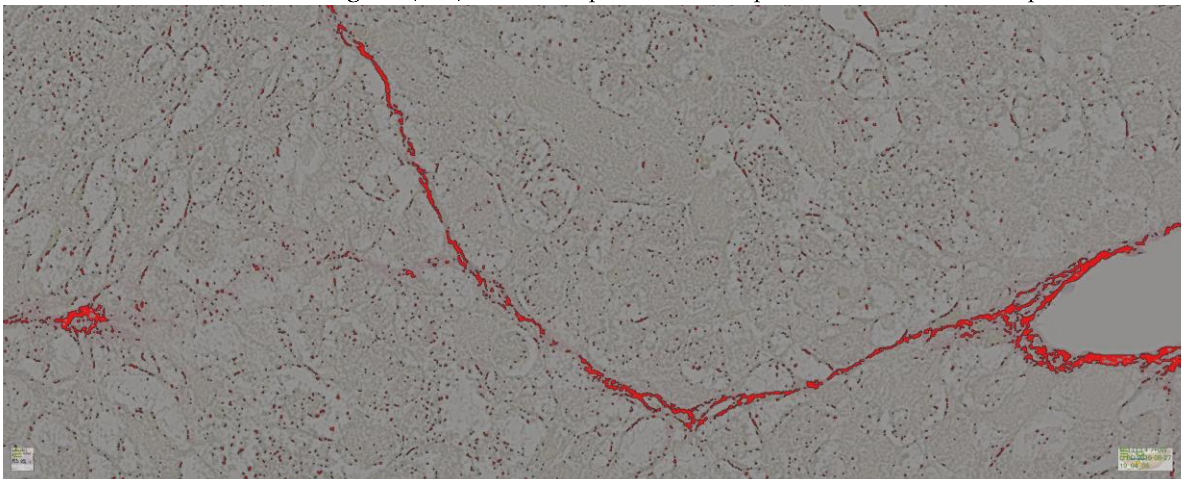

B. Optimized method

1. Pre-filters

a) RGB-G (mean)

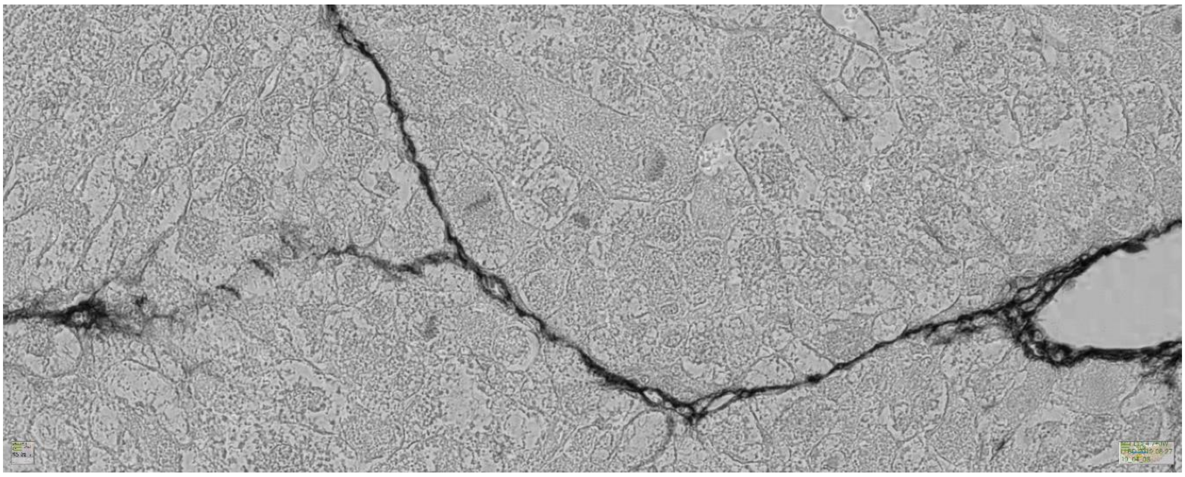

b) Hematoxylin / 3-Amino-9-Ethylcarbazole (H-AEC)

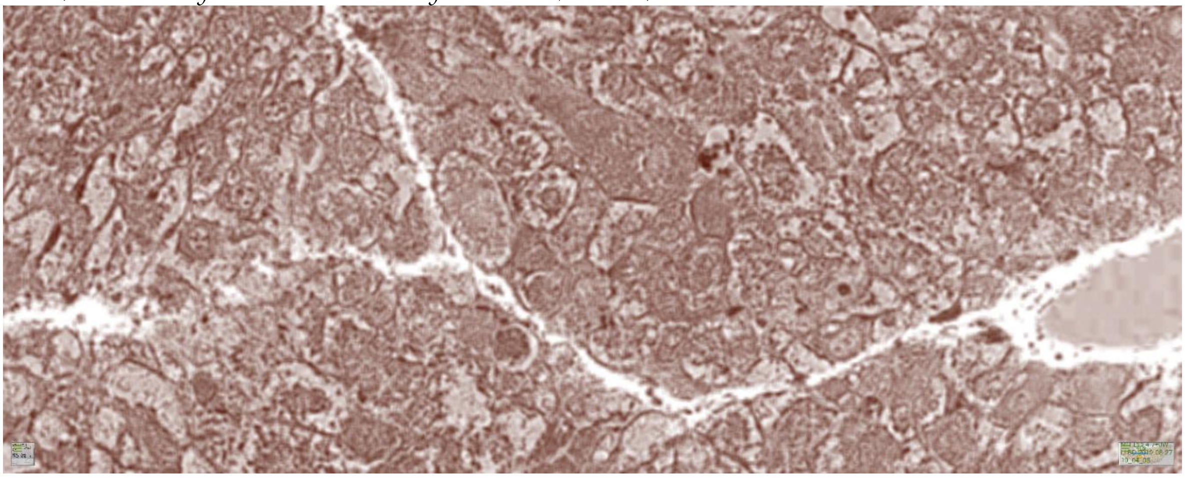

c) H-AEC divided by RGB-G (mean); noise filter

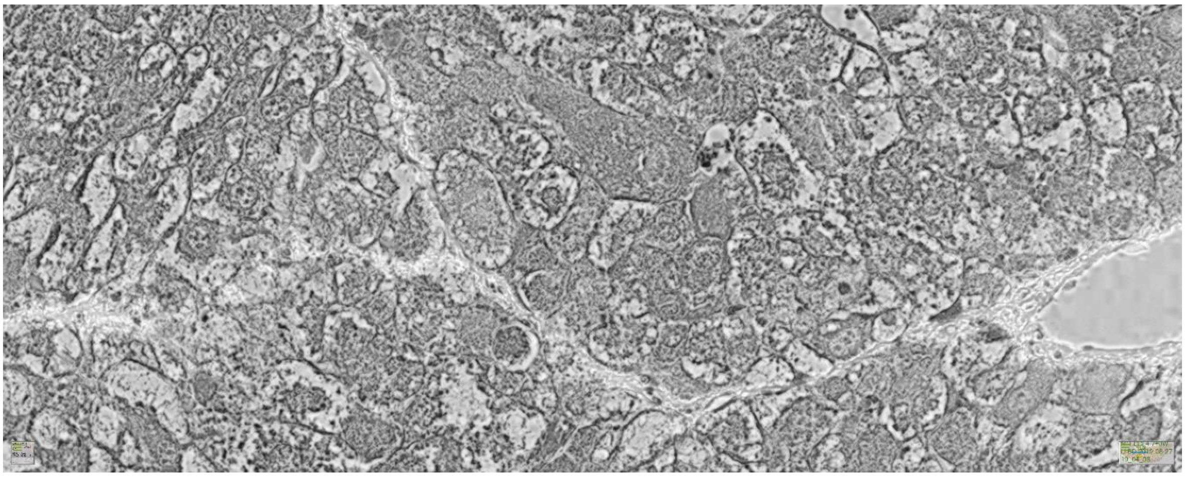

2. Detection filters

a) Red-Green Contrast filter minus noise filter

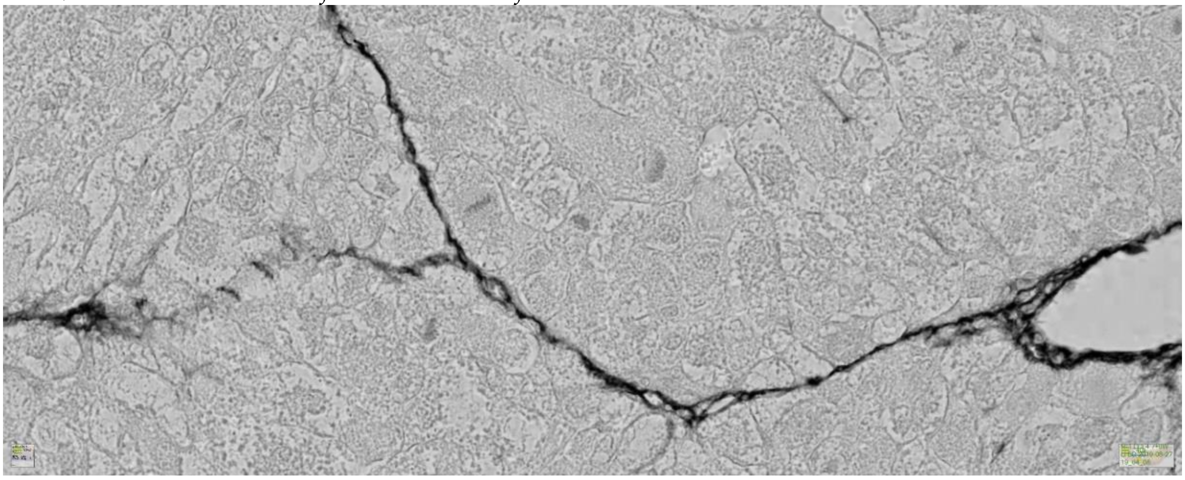

b) Chromaticity Red

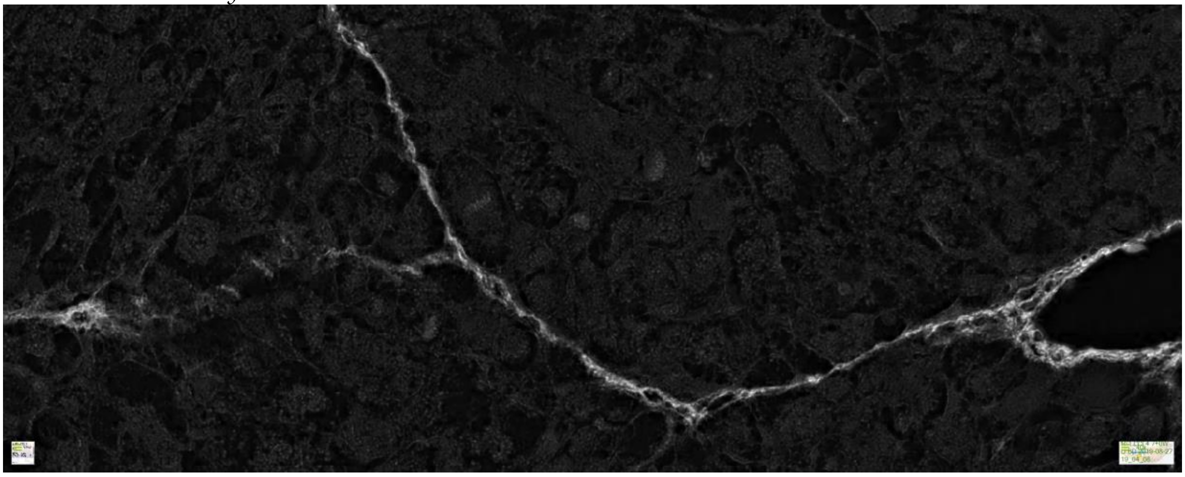

3. Result with PSR optimized method

Threshold set to best fit all organs (= 90). Note the good sensitivity (correct detection of faintly stained pixels) and specificity (no unstained pixel included) with these parameters. An example is given in the three studied organs in **Supplement material 3**. If needed, threshold can easily be adjusted per organ according to specific background. See below the great dynamic range of this method.

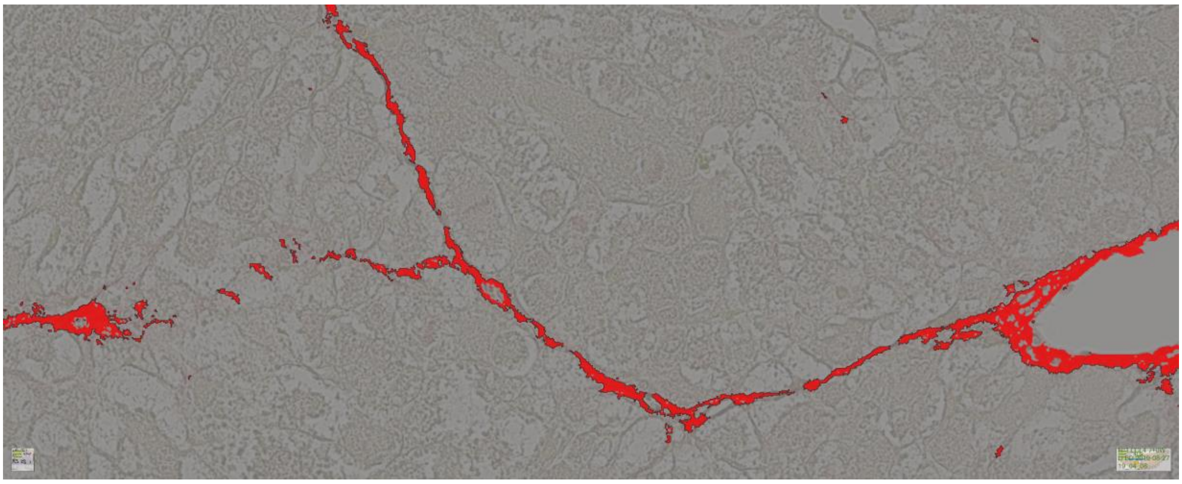

4. PSR optimized method: subsequent fiber classification

Having detected PSR staining, subsequent segmentation was achieved by watershed (Chromaticity red used as reference) and separate objects functions (size). Each segment was classified as *scattered fibers* (blue) or *compact fibers* (red) based on the mean intensity.

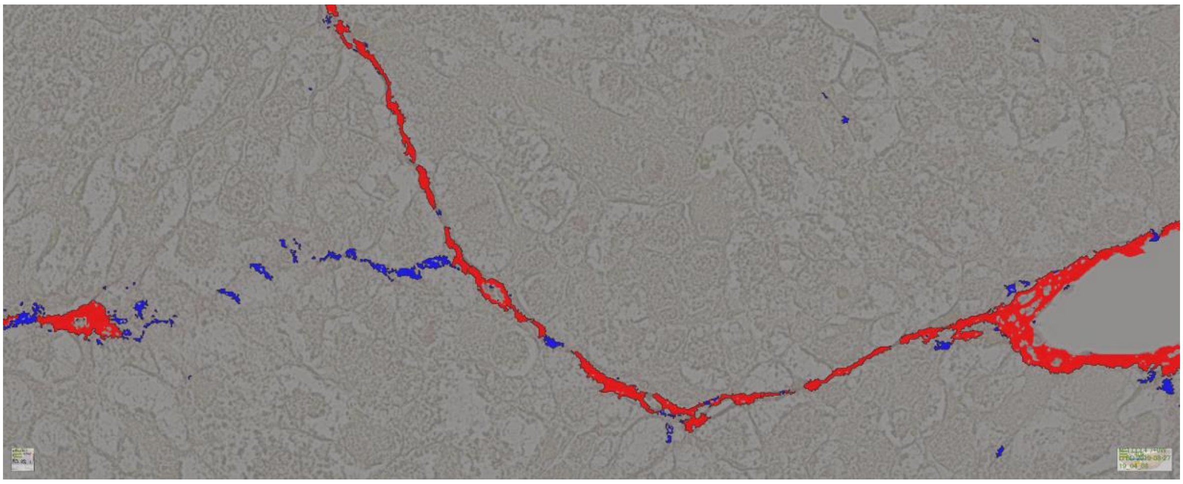

C. Dynamic range of the two detection methods

A. RGB method

threshold = 5

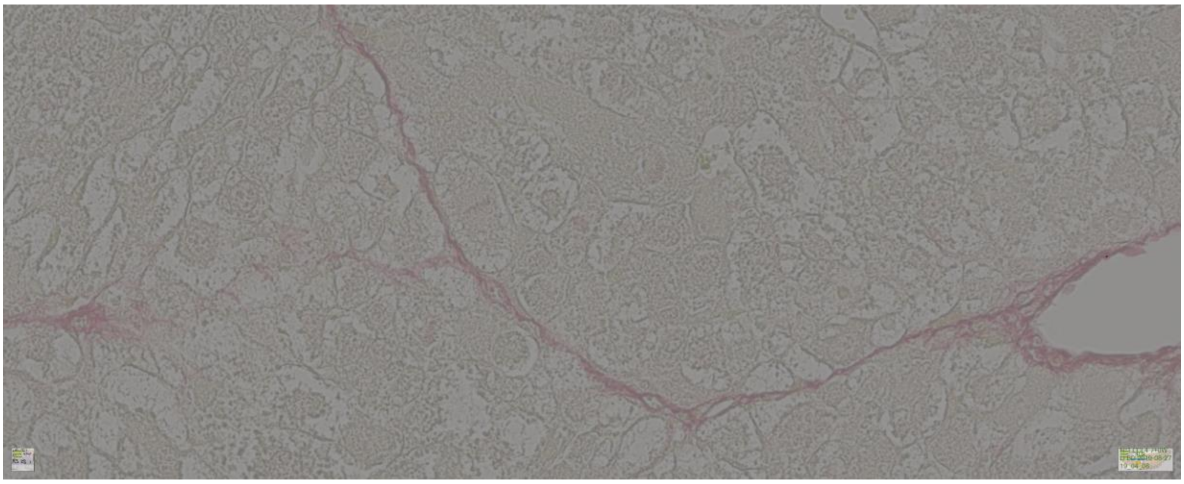

threshold = 20

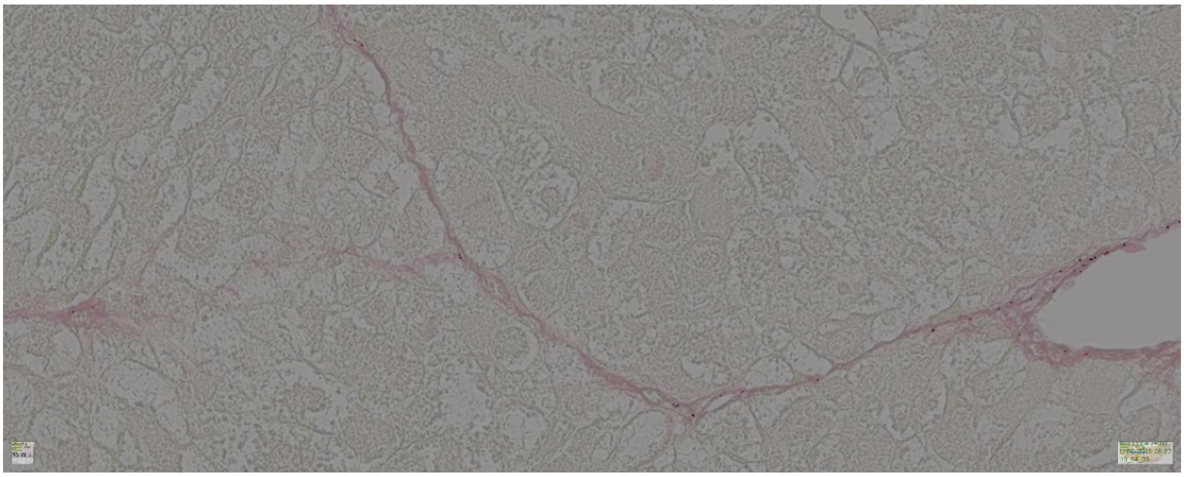

threshold = 40

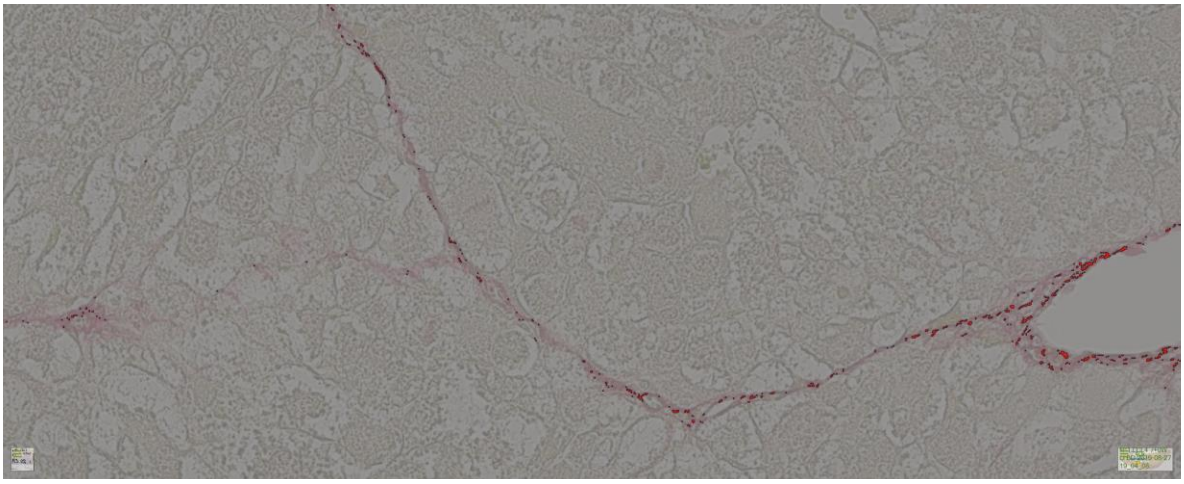

threshold = 50

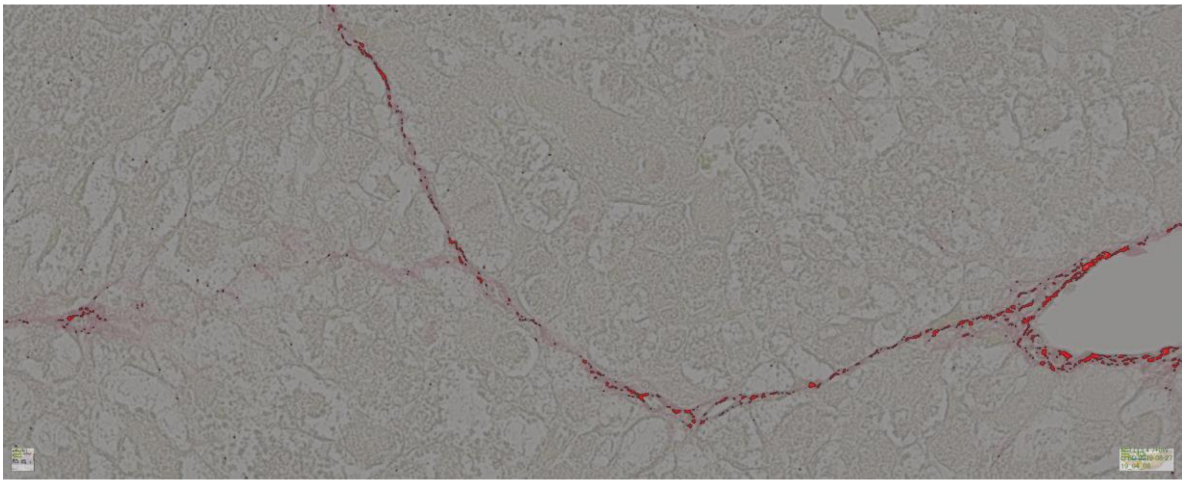

threshold = 60

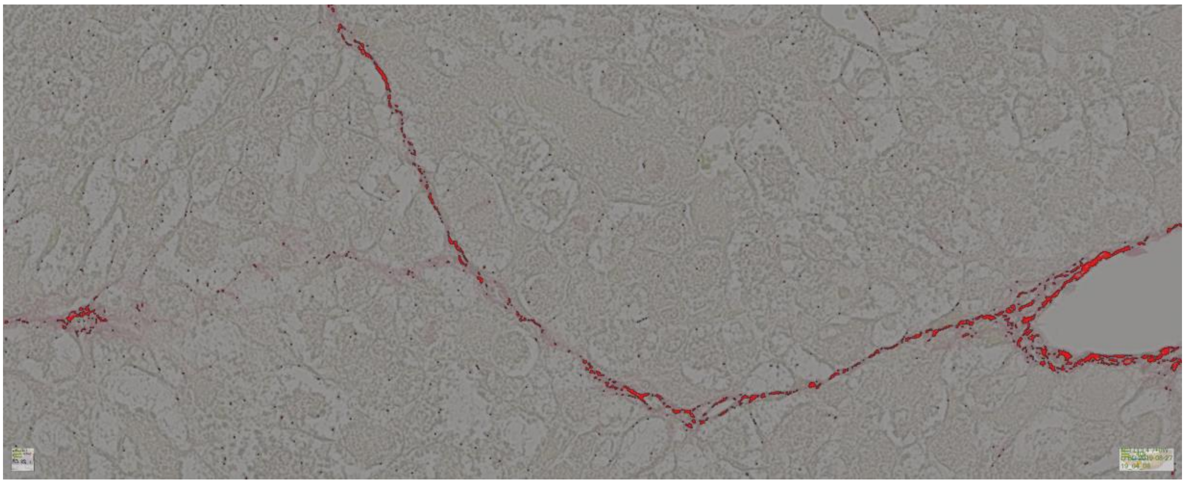

threshold = 70

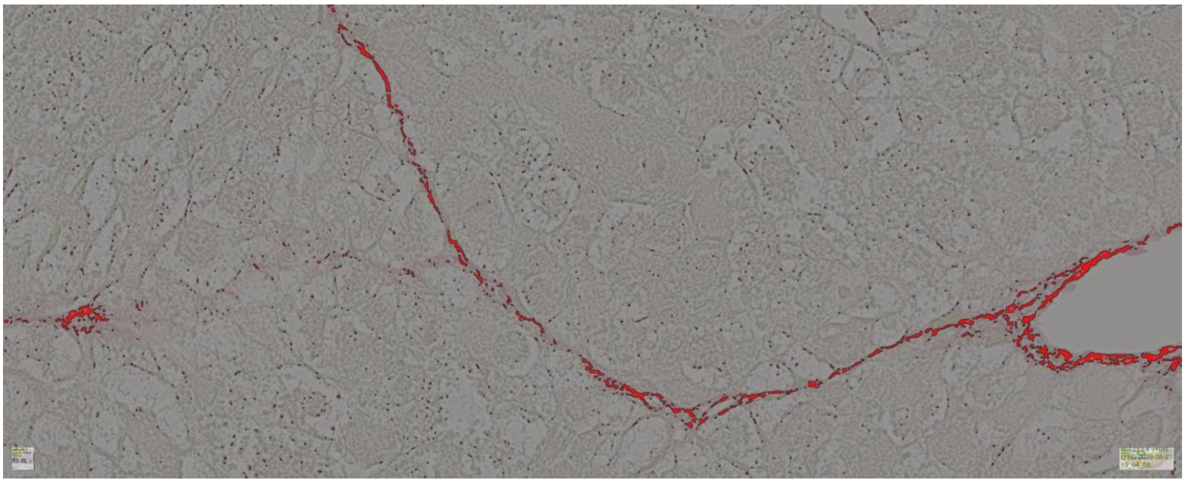

threshold = *80*

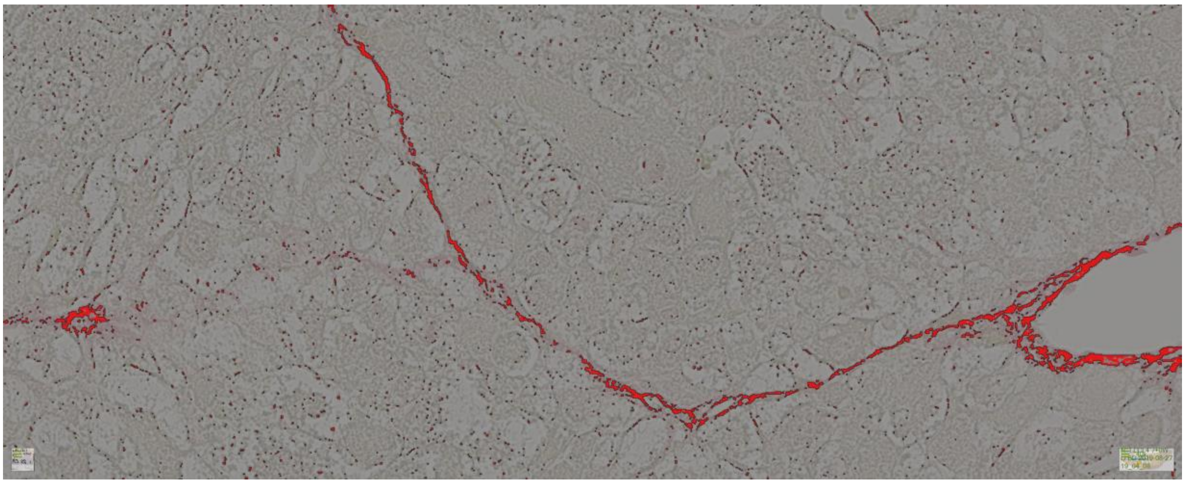

threshold = *85* ***(chosen for multi-organ comparison, Supplement material 3)***

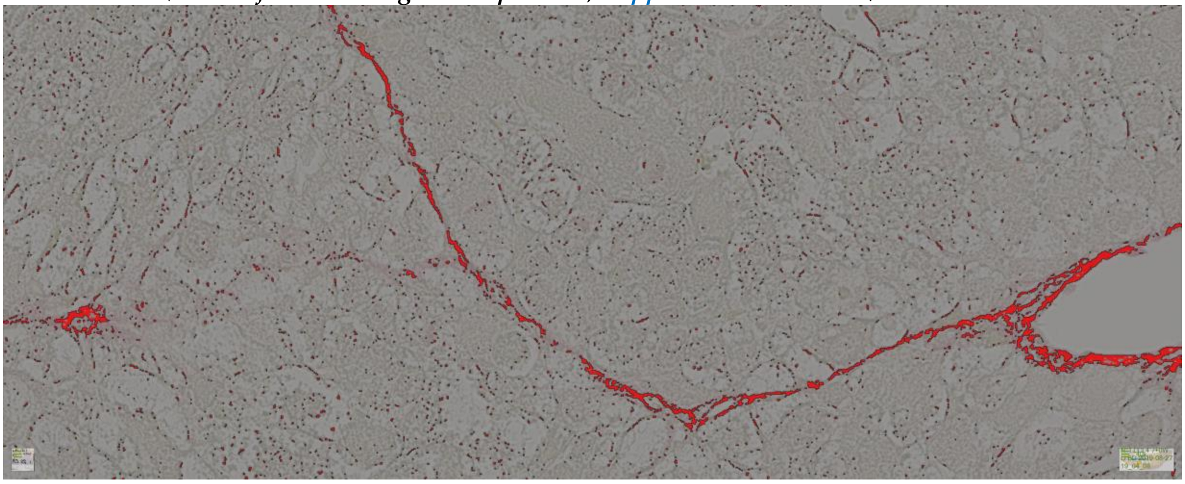

threshold = *90*

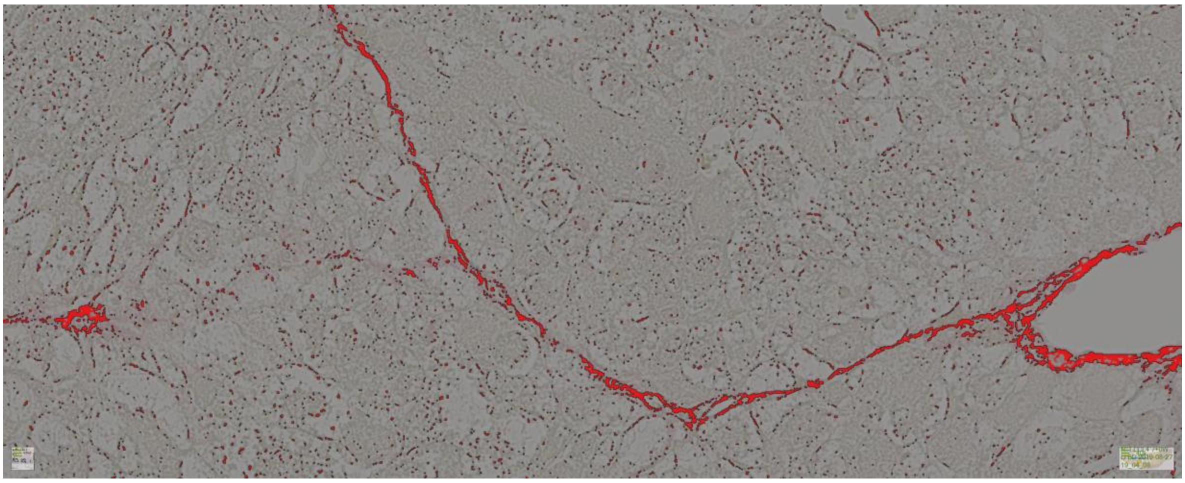

threshold = 100

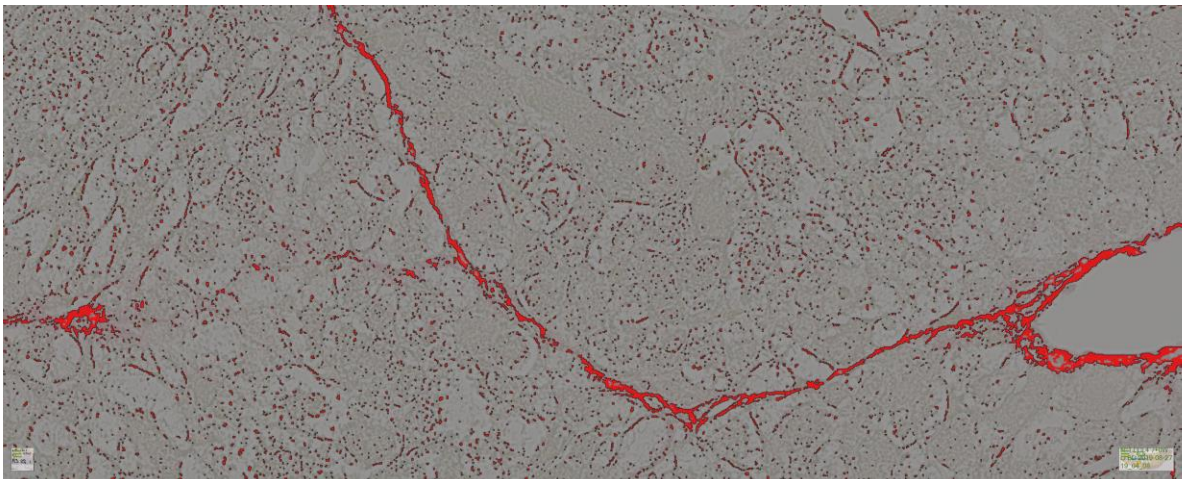

threshold = 120

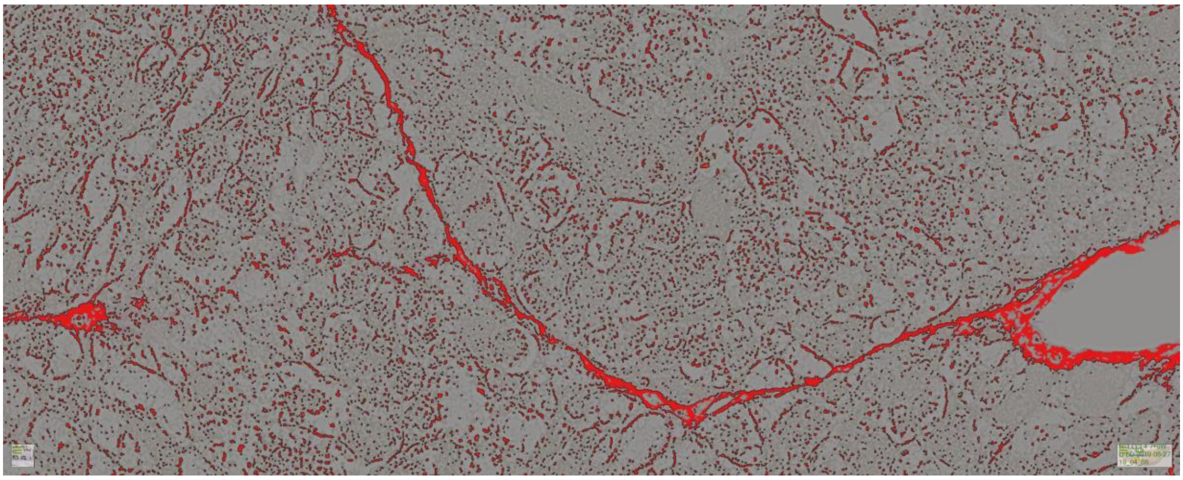

threshold = 140

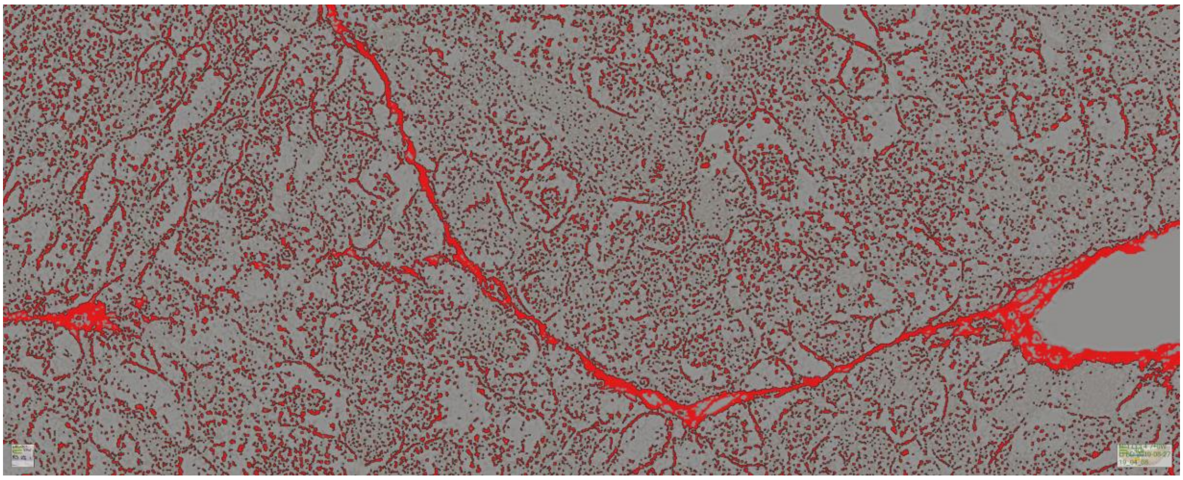

B. PSR optimized method

threshold = -20

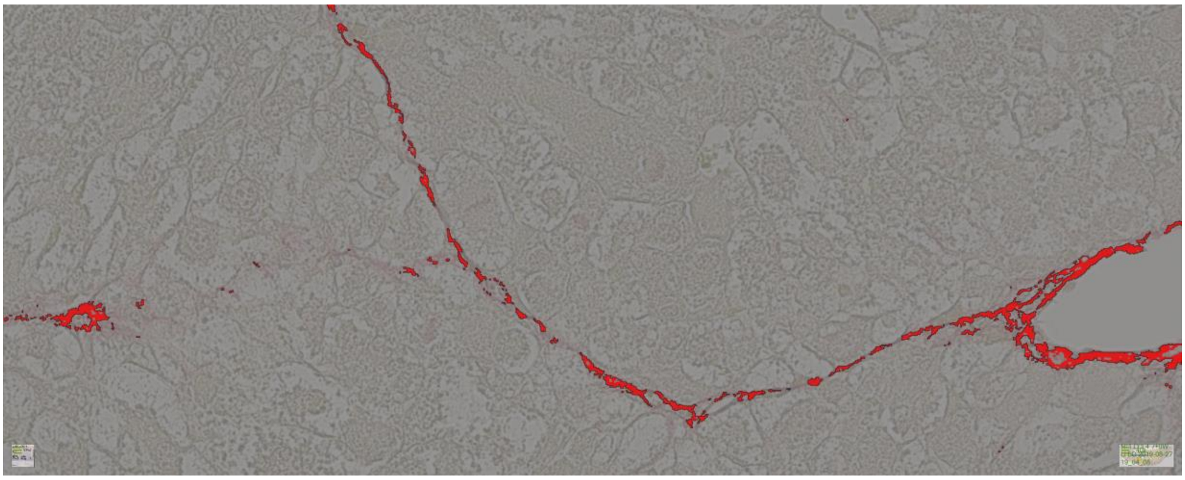

threshold = 0

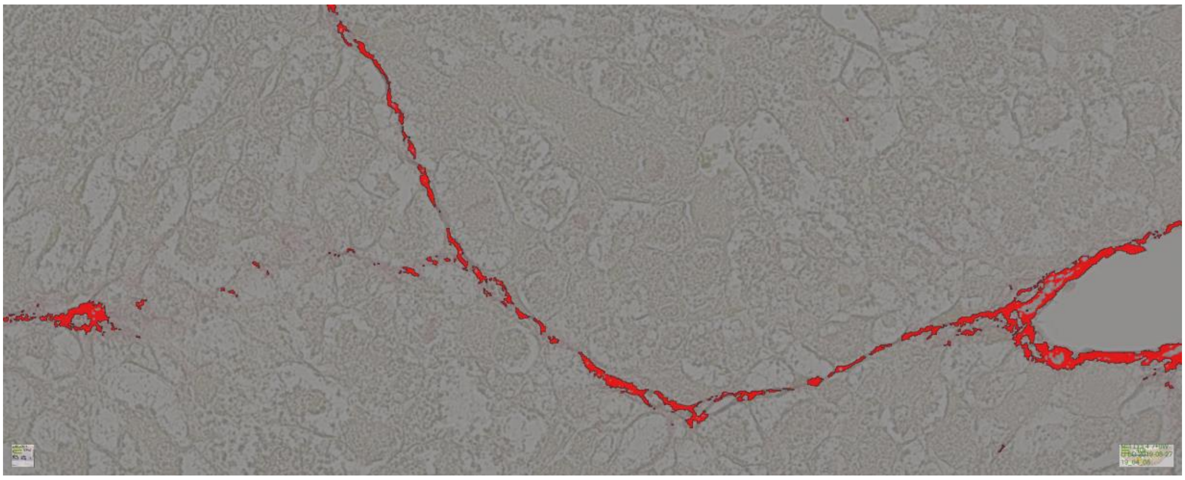

threshold = 20

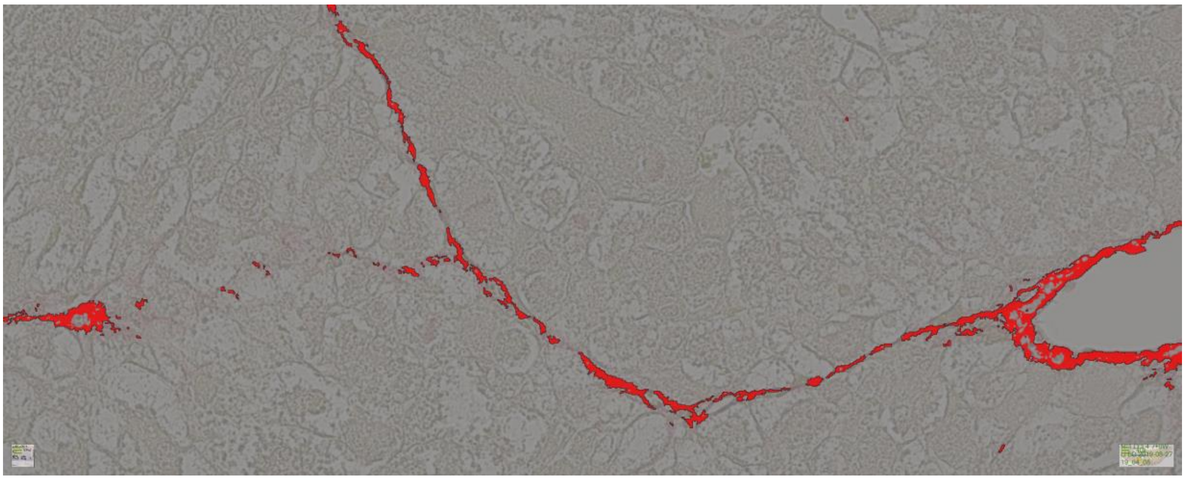

threshold = 30

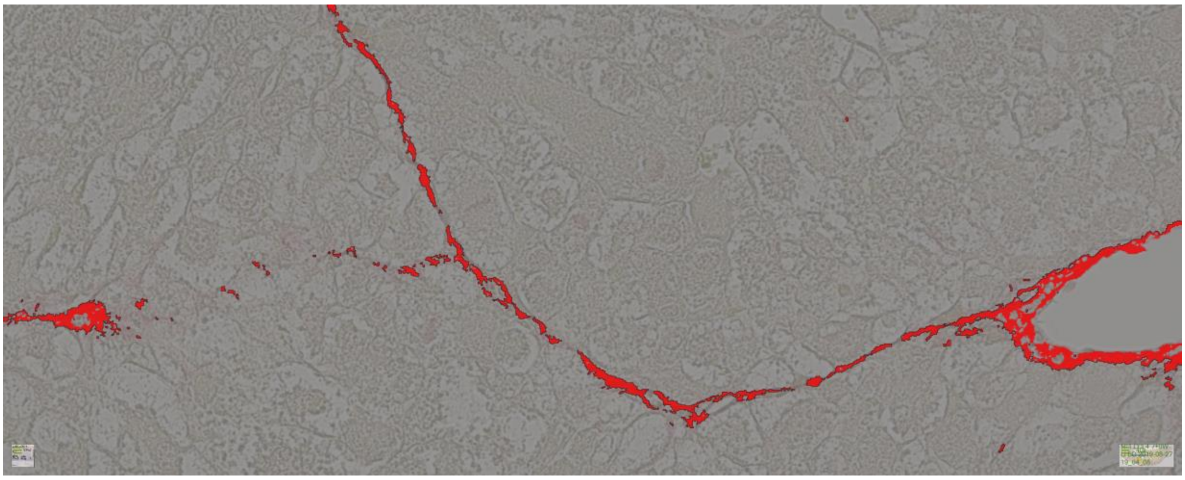

threshold = 40

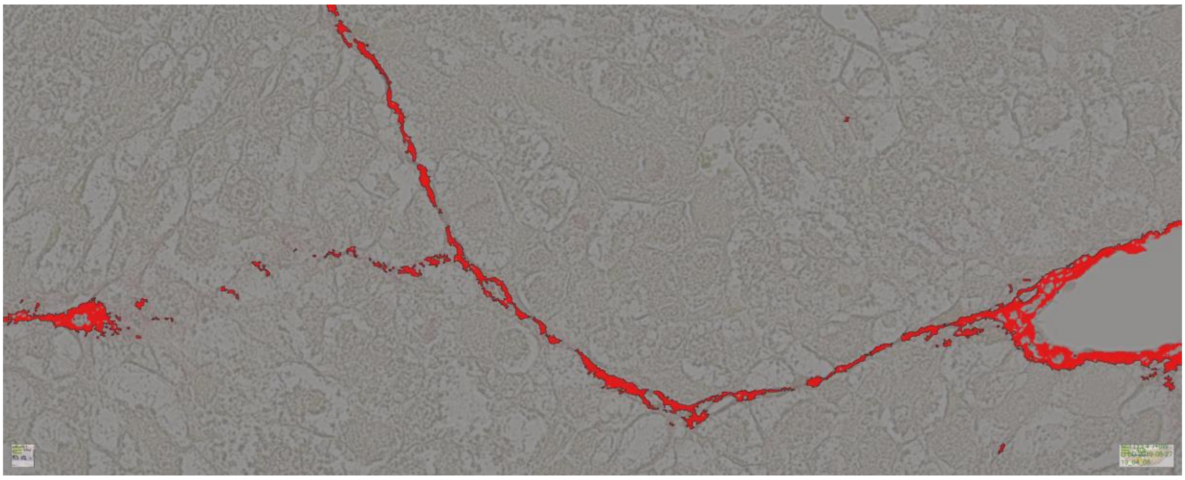

threshold = 50

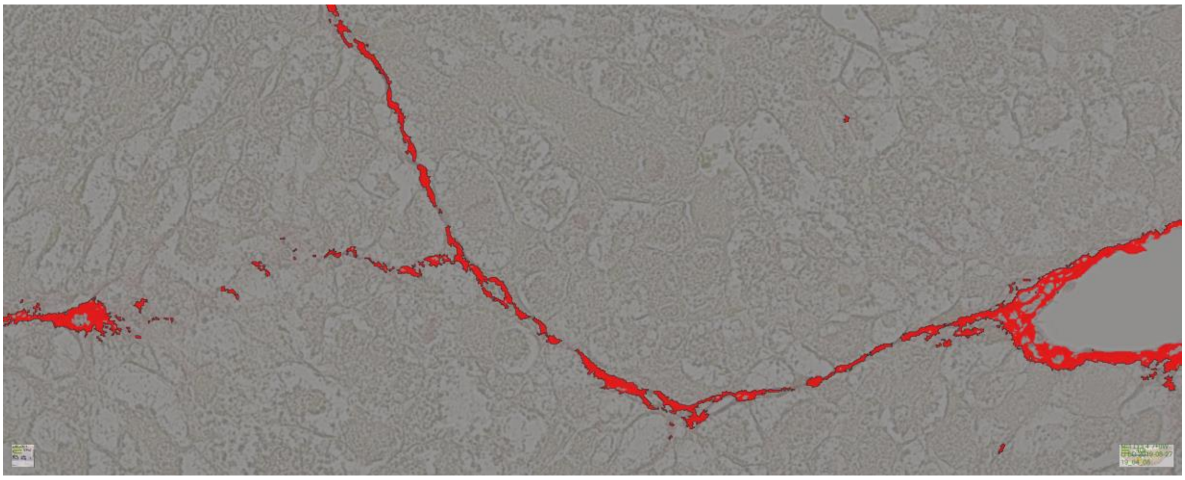

threshold = 60

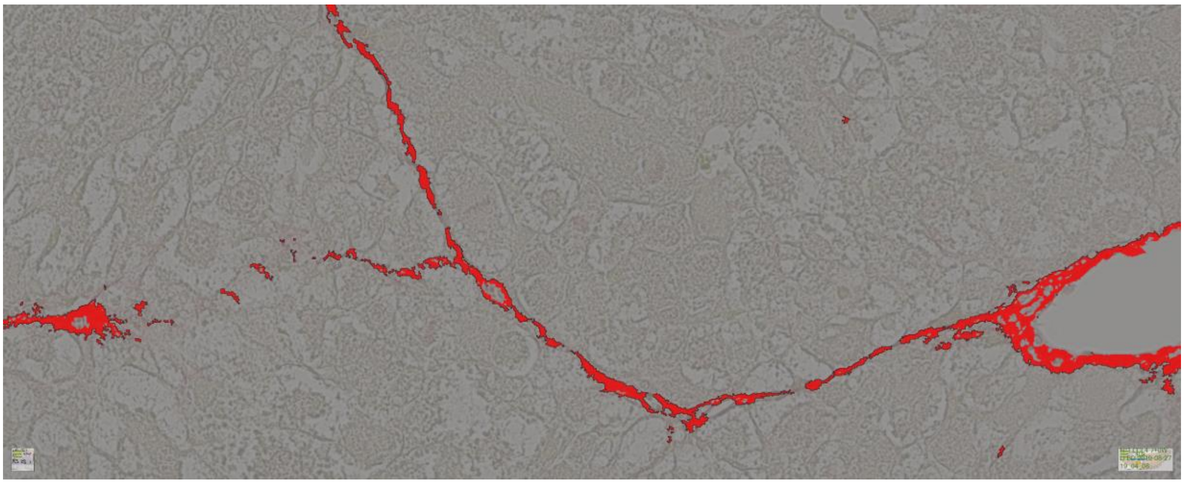

threshold = 70

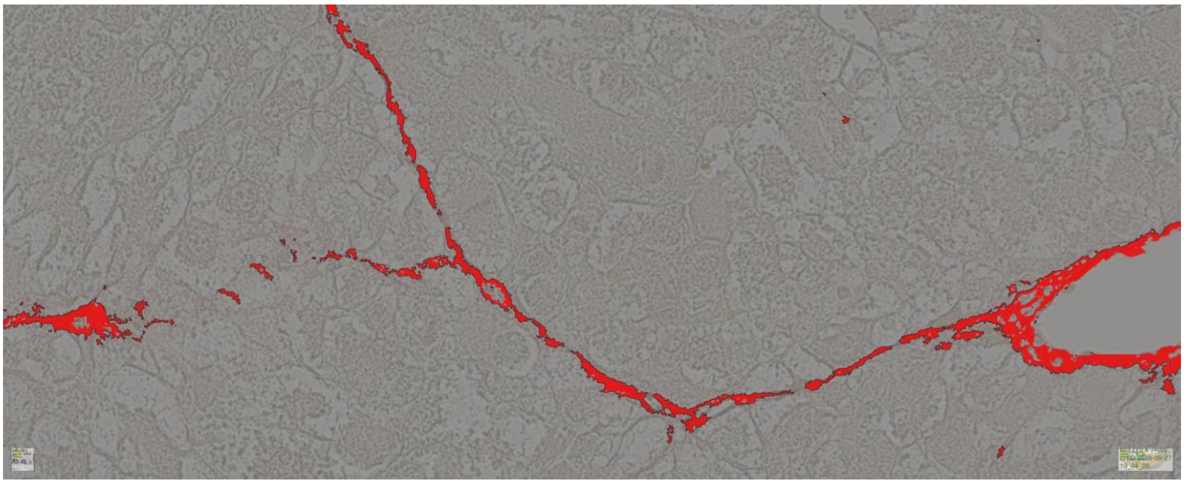

threshold = 80

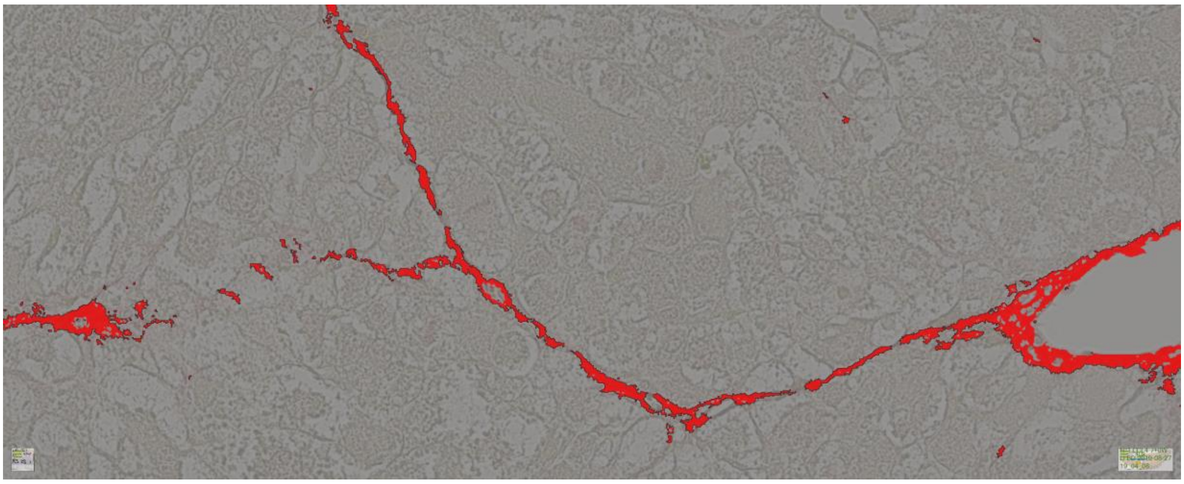

threshold = 90 ***(chosen for multi-organ comparison, Supplement material 3)***

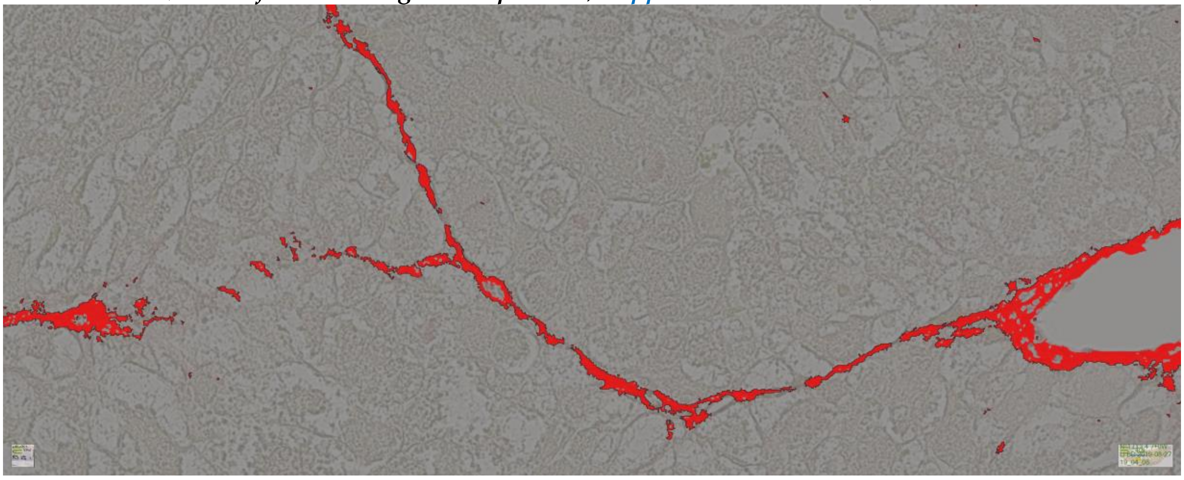

threshold = 100

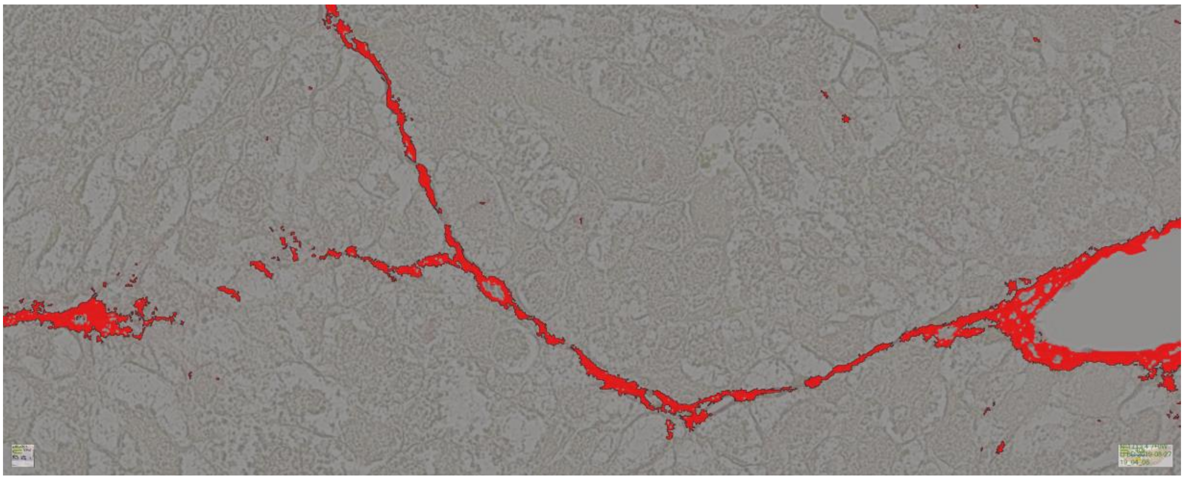

threshold = 110

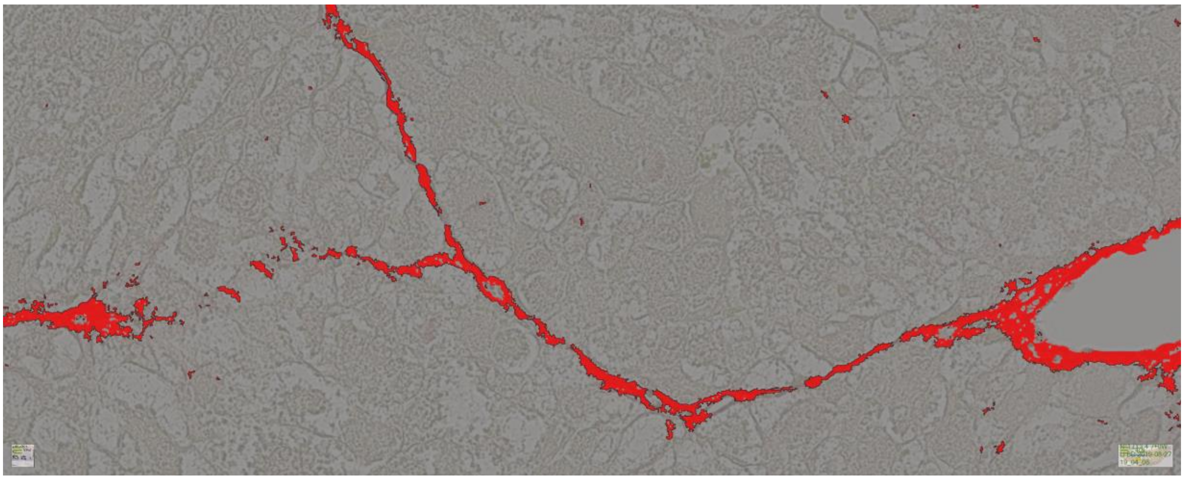

threshold = 120

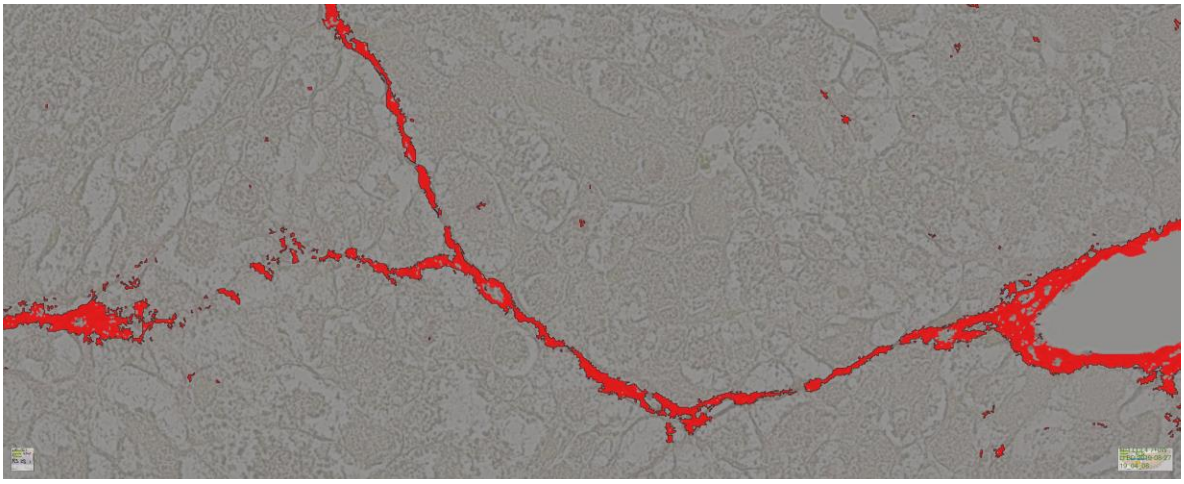

threshold = 130

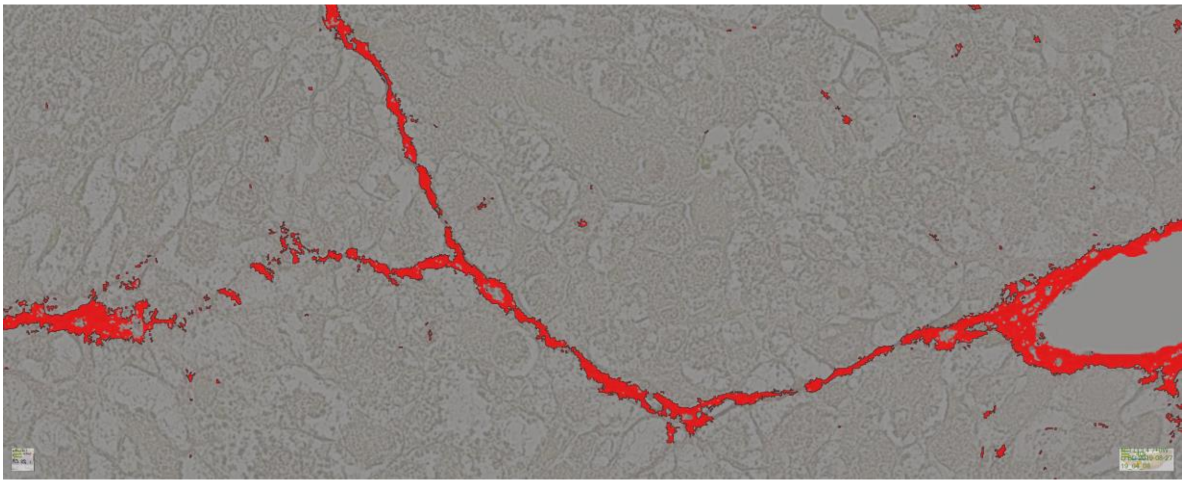

threshold = 140

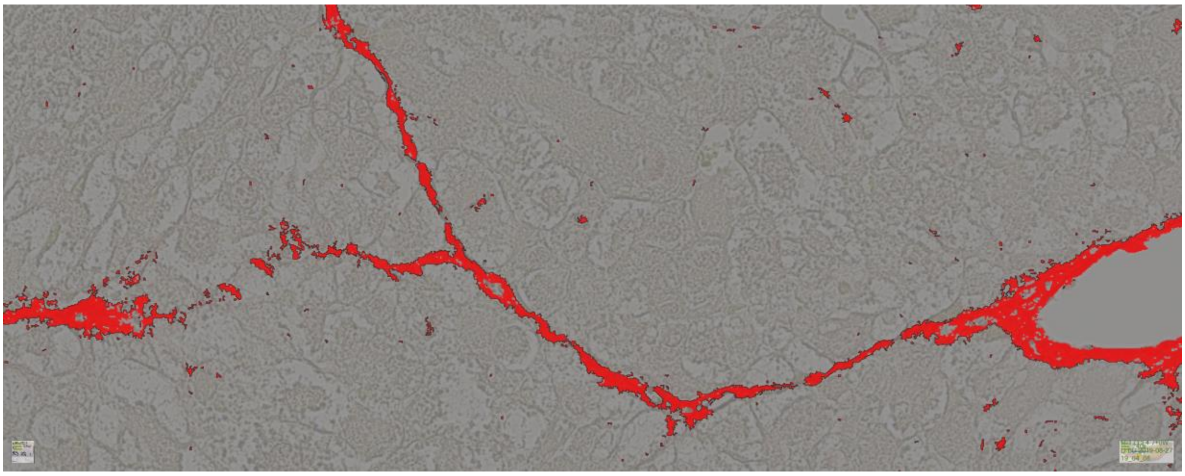

D. Comparison of the detection methods

An example is given for the detection of discrete liver fibrosis. Signal is either detected using the PSRRGB or the PSROPT filtering. Using a common threshold defined to best fit all organs, PSRRGB provided incomplete (yellow arrow) or irrelevant results (white arrow) (**B**). The accuracy of detection was improved with PSROPT method (**C**). Based on PSROPT detection method, it was possible to segment the fibers and to apply an automated classification based on the staining intensity and pixel homogeneity (**D**).

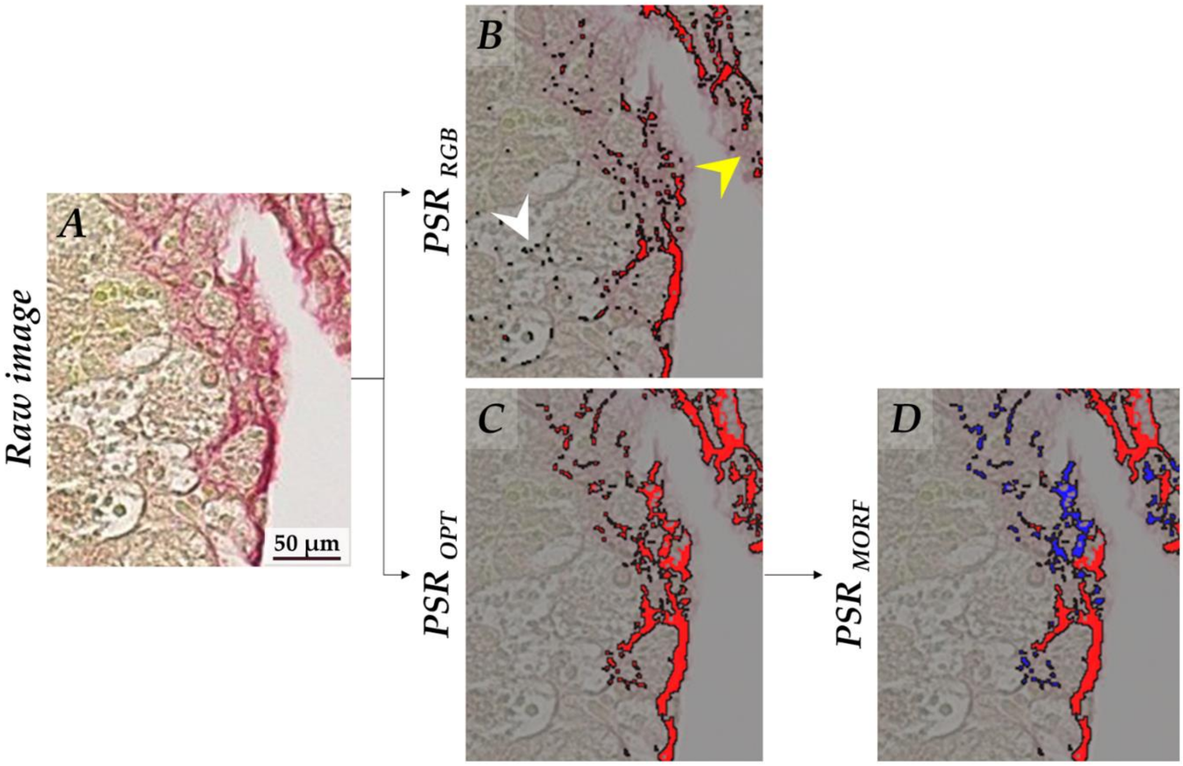

Supplement: Supplementary file 1 [file biomolecules-10-01585-s001.zip › Courtoy et al. Supplementary Material 2.pdf]
