# Supplementary material for: Digital Image Analysis of Picrosirius Red Staining: A Robust Method for Multi-Organ Fibrosis Quantification and Characterization
Source: Biomolecules. 2020 Nov 22;10(11):1585. doi: 10.3390/biom10111585 (PMC7709042; doi:10.3390/biom10111585)
Supplement: Supplementary file 1 [file biomolecules-10-01585-s001.zip › Courtoy et al. Supplementary Material 3.pdf]

Supplementary Material 3: RGB-based and the PSR-optimized methods used for multi-organ comparison in liver, lung and kidney (illustration and correlation).

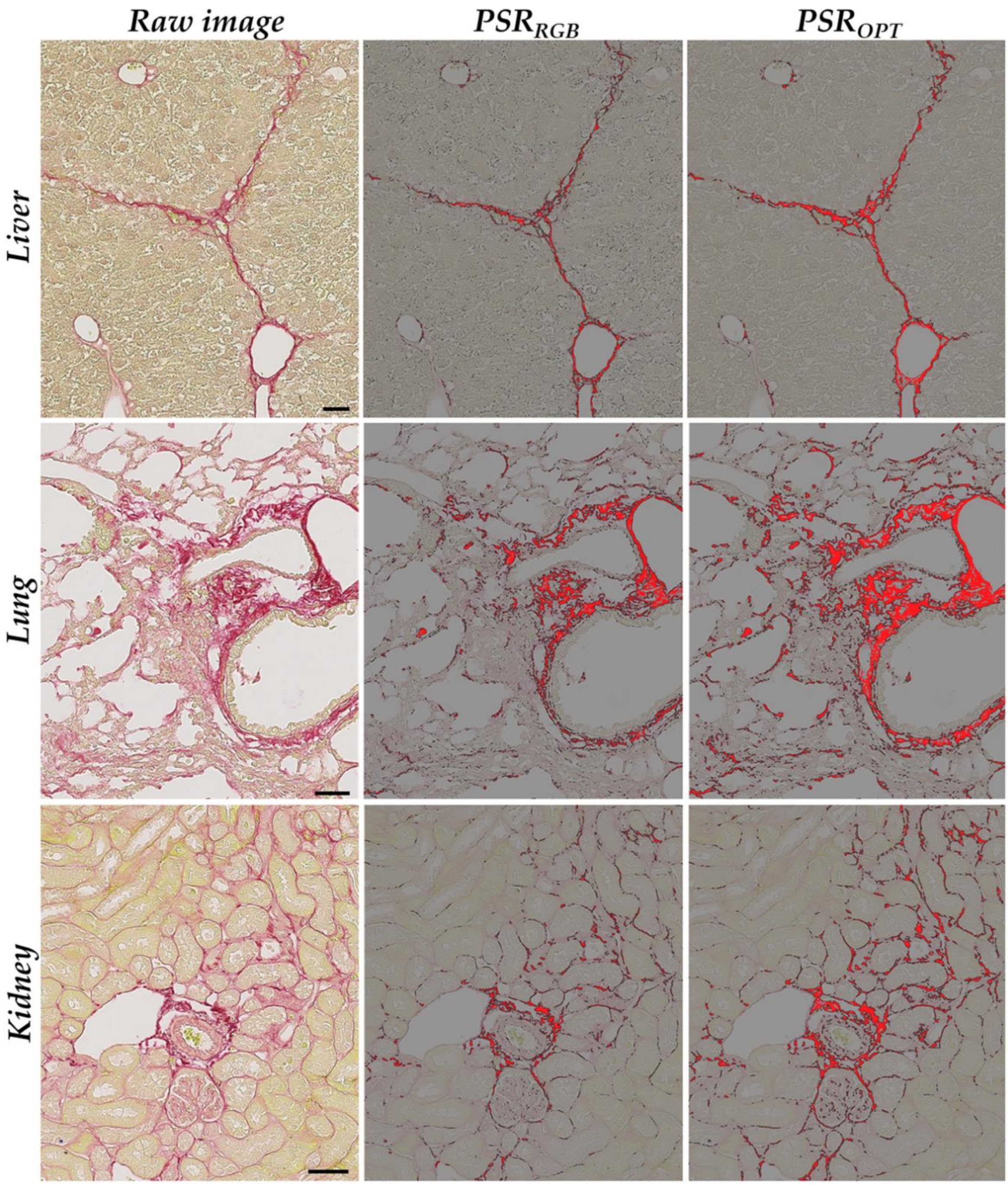

3A. The RGB-based method (PSR_{RGB}) performed fairly well to detect large collagen fibers but faint staining remained undetected whereas unsaturated pixels were considered positive. These errors were not comparable between organs. Indeed, the normal hepatic parenchyma showed frequent unsaturated pixels, which is less the case in the two other organs. The same threshold was set by empirical adjustment to fit the three models. The PSR_{OPT} method was optimized to limit these errors by exclusion of unsaturated pixels and better detection of faintly stained pixels than done by RGB filtering. The same threshold was set by empirical adjustment to fit the three models. Of note, model-specific threshold could be easily adjusted. Scale bar = 50 µm

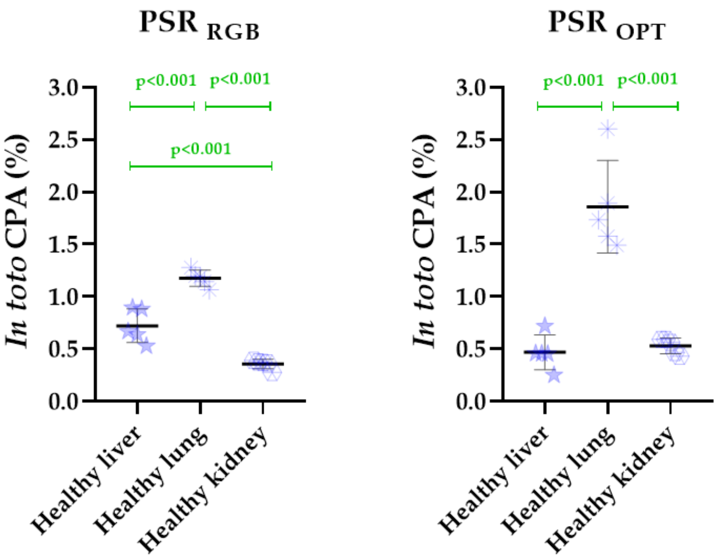

3B. Using the PSR$_{RGB}$ and PSR$_{OPT}$ algorithms with the empirically defined common threshold for each detection method, it was possible to compare PSR staining in the three organs. The comparison is shown here for the controls (considered healthy status) and as *in toto* values (all tissue except the capsule or the pleura). Both methods showed that, in average a healthy lung displays more extended stained area than healthy liver and kidney.

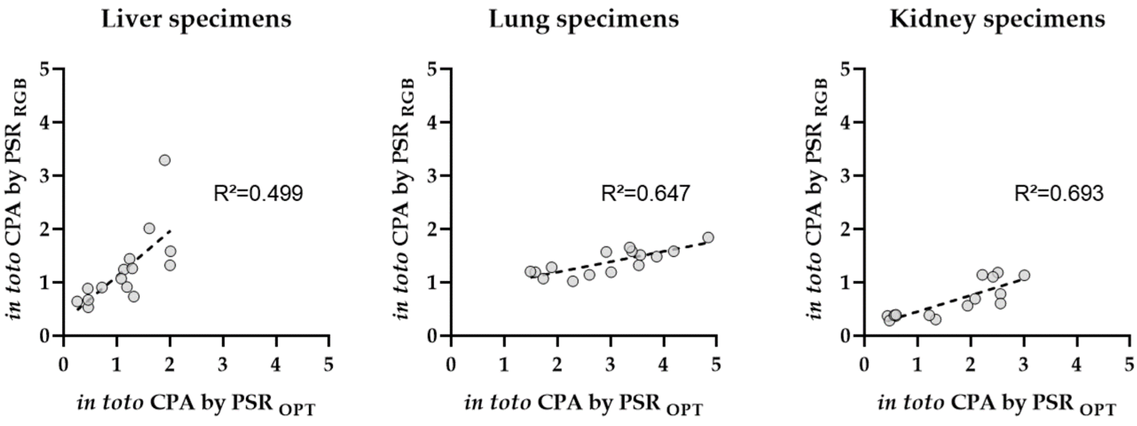

3C. Focusing on each model, the coefficient of goodness-of-fit was of 0.499 for the livers, 0.647 for the lungs and 0.693 for the kidneys, respectively. The lower reproducibility in the liver compared to the other organs was attributed to lower signal-to-noise ratio using PSR_{RGB} in the liver, and therefore more aspecific signal. In the lung and kidney, the coefficient of correlation was superior, while CPA values obtained with PSR_{OPT} were higher than using PSR_{RGB} detection method.
