# Supplementary material for: Digital Image Analysis of Picrosirius Red Staining: A Robust Method for Multi-Organ Fibrosis Quantification and Characterization
Source: Biomolecules. 2020 Nov 22;10(11):1585. doi: 10.3390/biom10111585 (PMC7709042; doi:10.3390/biom10111585)
Supplement: Supplementary file 1 [file biomolecules-10-01585-s001.zip › Courtoy et al. Supplementary Material 4.pdf]

Supplementary Material 4: Validation of the developed PSR-optimized method vs. previously described PSR-detection method on murine kidney samples.

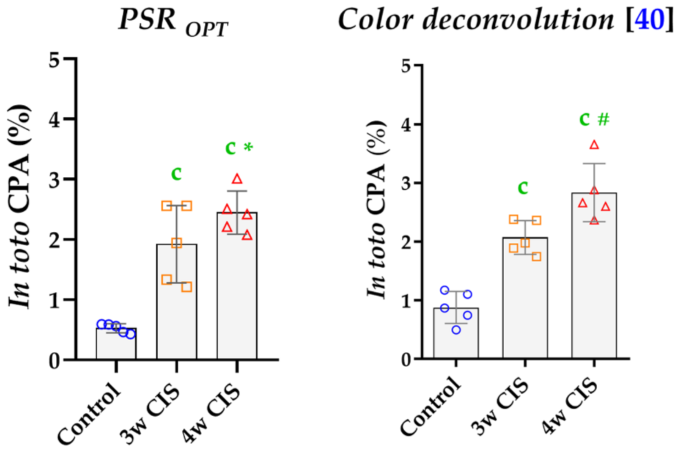

4A. The PSR$_{OPT}$ method was optimized to suit on three different organs and a unique threshold was set to detect fibrosis for all the models. To challenge its validity, this method was compared to the gold-standard method for renal fibrosis, based on a color deconvolution of the signal and an organ-specific threshold (Bienaime *et al.* 2016, [40]). *In toto* CPA values obtained with both methods showed very similar ranges, demonstrating therefore the robustness of PSR$_{OPT}$ method. c: $p<0.001$ vs. controls, *:$p<0.050$ vs. 3w CIS, #:$p<0.010$ vs. 3w CIS.

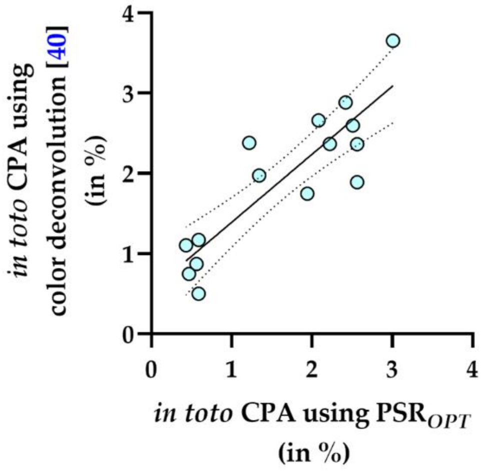

4B. Linear regression of *in toto* CPA values obtained with PSR$_{OPT}$ and color deconvolution (Bienaime *et al.* 2016, [40]) methods on all kidney samples. Correlation was excellent between the two methods (Person $r = 0.874$; $p<0.001$) and data followed a linear trend (goodness-of-fit, $R^2 = 0.764$).
